# Evaluation of imputation strategies for multi-centre studies: Application to a large clinical pathology dataset

**DOI:** 10.1371/journal.pone.0335852

**Published:** 2025-11-20

**Authors:** Lucy Grigoroff, Reika Masuda, John Lindon, Janonna Kadyrov, Jeremy K. Nicholson, Elaine Holmes, Julien Wist

**Affiliations:** 1 Australian National Phenome Centre, and Centre for Computational and Systems Medicine, Health Futures Institute, Murdoch University, Perth, Australia; 2 Institute of Global Health Innovation, Faculty of Medicine, Imperial College London, London, United Kingdom; 3 Department of Metabolism, Digestion and Reproduction, Faculty of Medicine, Imperial College London, London, United Kingdom; 4 Chemistry Department, Universidad del Valle, Cali, Colombia; Max Planck Institute for Solid State Research, GERMANY

## Abstract

As part of a strategy for accommodating missing data in large heterogeneous datasets, two Random Forest-based (RF) imputation methods, missForest and MICE were evaluated along with several strategies to help navigate the inherently incomplete structure of the dataset. Background: A total of 3817 complete cases of clinical chemistry variables from a large-scale, multi-site preclinical longitudinal pathology study were used as an evaluation dataset. Three types of ‘missingness’ in various proportions were artificially introduced to compare imputation performance for different strategies including variable inclusion and stratification. Results: MissForest was found to outperform MICE, being robust and capable of automatic variable selection. Stratification had minimal effect on missForest but severely deteriorated the performance of MICE. Conclusion: In general, storing and sharing datasets prior to any correction is a good practise, so that imputation can be performed on merged data if necessary.

## Introduction

Advances in high-throughput technologies have enabled the generation and accumulation of large-scale biological and chemical datasets that are increasingly applied in molecular epidemiology studies. Datasets encompassing diverse data types, including genome sequences, proteomic profiles, and compound libraries can contribute to our understanding of complex biological phenomena, disease mechanisms, ecological community structures and drug discovery processes. However, missing values are common in most large-scale multiparametric studies and this inherent incompleteness presents significant informatic and modelling challenges. Incompleteness results from a variety of diverse factors and experimental limitations, data acquisition errors, or the sheer scale of data collection efforts, often carried out across multiple centres or over disjointed time periods. For example, in clinical studies, the lack of standardisation in diagnostic reporting and medical records may result in missing clinical annotations, particularly when data collection is dispersed over multiple centres. Further, the range of recorded clinical annotations depends on the research question resulting in missing values when two or more studies are combined for analysis. Missing samples are also common in single cohort studies; It is likely that some samples will not be collected or that one of the many experimental steps from sample preparation to analysis will fail, thus producing incomplete datasets. Whether from the experimental data or clinical annotations (metadata), missing values can introduce different types of bias in the subsequent analysis or prevent application of certain approaches [1,2].

The presence of missing values poses a critical impediment to downstream analyses, including statistical analysis, machine learning, and predictive modelling. Missing data can introduce biases, reduce statistical power, and compromise the validity of biological and chemical inferences. Therefore, the development of robust imputation strategies is paramount to address these gaps, ensuring the integrity and enhancing the value of the datasets. Effective imputation not only enhances the quality and completeness of the data but also facilitates more accurate and reliable analyses, leading to deeper insights into biological mechanisms and chemical interactions.

Given the complexity and heterogeneity of biological and chemical data, the design of imputation methods tailored for studies collected longitudinally in batches is desirable. As the volume and diversity of omics and chemical data continue to expand, there is a need for sophisticated imputation approaches that minimise reprocessing as new data are appended.

Several multivariate analysis techniques, including Principal Component Analysis (PCA) and Partial Least Squares-Discriminant Analysis (PLS-DA) require a dataset to be complete, i.e., they do not accept missing values. Furthermore, as entire rows (samples) and/or columns (variables) are excluded, the statistical power of the study is reduced [3–5], and valuable information is lost. To overcome this deletion referred to as Complete Case Analysis (CCA) numerous imputation methods have been proposed that replace missing values with imputed ones.

There is no strong consensus as to which single imputation method is best or which performance metrics are most appropriate; The method adopted is highly dependent on the dataset, *a-priori* knowledge and the causes behind missing values. If the reason for a data point being absent is independent of the data, it is referred to as Missing Completely At Random (MCAR). If the probability of a data point being absent is dependent on a known variable, it is referred to as Missing At Random (MAR). Where the probability of a point being absent is unexplained and does not fit the criteria for MCAR or MAR is referred to as Missing Not At Random (MNAR). Some methods are more suitable for certain types of missingness, and some methods become ineffective once a certain proportion of missingness is reached [6–10]. Furthermore, not all methods are suitable for categorical and/or continuous variables. The choice of method is context dependent; Sophisticated methods such as Parametric Multiple Imputation by Chained Equations (MICE) cannot account for variable interactions, non-linearities and non-normality without accurate and detailed specifications in the hyperparameters or pre-imputation transformation of the data [7,11–20].

**Fig 1:**
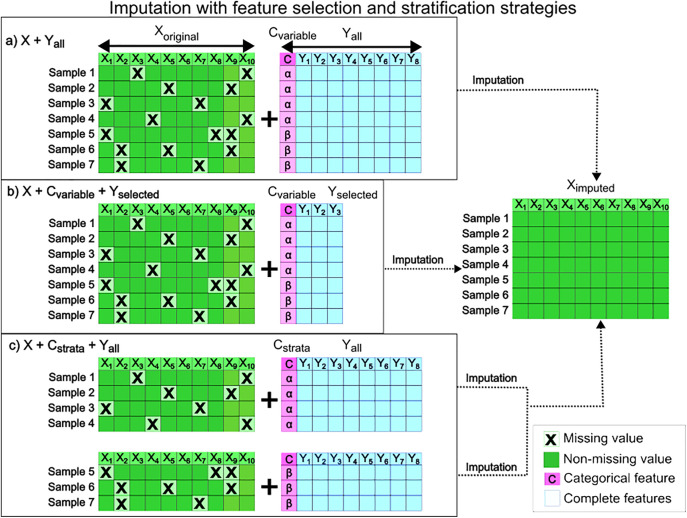
Schematic of different imputation strategies. X_Original_ stands for a block of independent variables (features) with missing values marked by an **x.** The Y block contains outcome parameters (metadata, or clinical annotations), with column C being the outcome variable of interest reflecting the experimental design with treatment status α or β. Block X_imputed_ is block X after imputation. In a) and b) imputation includes C as a variable, while in c) data for treatment status α and β are imputed separately and joined afterward.

Utilising *a-priori* knowledge about covariates or experimental design is important to avoid introducing unnecessary bias in the imputed dataset, and consequently in future analysis [21]. This can be achieved in two ways, by appending this extra information to the dataset or by stratifying the dataset accordingly. Let’s consider an outcome variable (column C in Fig 1) that contains, for example, a treatment or control status that accepts two values α and β. This outcome variable reflects the study experimental design and thus the known part of the inherent structure in the data. Including C as a feature (variable) is demonstrated in Fig 1a and 1b, while performing separate imputation models per strata defined by C is depicted in Fig 1c [5,22]. While it is common practice to include all available features as input (Fig 1a), this can lead to overparameterization when the number of variables exceeds the number of samples (p > n) [23]. Therefore, some imputation methods, such as Random Forest (RF), include a feature selection procedure [24]. This feature selection may result in the exclusion of one or more columns of Y, as depicted in Fig 1b. The impact of including the outcome variable column C in Fig 1, on the accuracy of the imputation has been discussed for machine learning methods such as MICE [25].

A notable gap in the current literature is the comparison between the two strategies, stratified imputation (Fig 1c) or inclusion of outcome variables in the imputation process (Fig 1a and 1b), with or without with feature selection (Fig 1b) [22,26–29]. In this study we set out to identify a suitable implementation of a practical and robust imputation method for datasets resulting from the analysis of multiple epidemiological studies or from datasets across multiple national databanks.

As an example, we evaluate different strategies to impute a large-scale chemical pathology dataset, using a variety of performance metrics for accuracy and precision, to select the methods that best maintains data integrity. This dataset was generated from the COnsortium for MEtabonomic Toxicology (COMET), a large multi-centre preclinical toxicology study in a rat model, aimed at building augmented models of the toxicity for a range of compounds. Within this project, all the data were generated and recorded using a harmonised protocol across five different pharmaceutical companies [30–32]. The dataset consists of serum clinical chemistry measurements for a range of parameters typically used in acute toxicity studies aiming to assess the safety of drug candidates. Data were generated for 21 serum parameters measured for 107 chemical pathogens (86) or physiological stressors (21) across a dataset of 7528 samples. This dataset contains inherent structure, such as samples being collected from different companies with different suppliers, for different toxins, and for high- low-dose and control groups and provides a good example of the challenges of joining multiple epidemiological studies for the construction of atlases of diseases, and more generically large biological and chemical datasets.

## Methods

### Dataset

**Fig 2:**
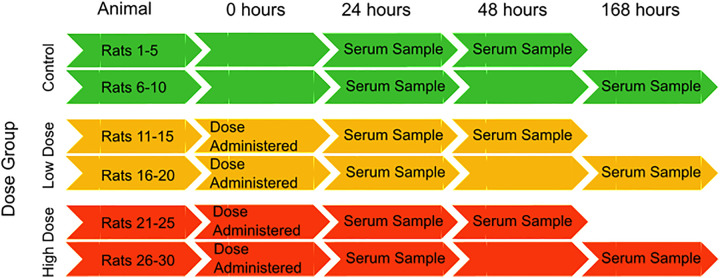
Serum sample collection details for all COMET studies. Each study had 30 rats assigned across control, low dose and high dose with only two serum samples per rat.

The COMET project, a preclinical rat toxicology study, resulted from a collaboration between five pharmaceutical companies and Imperial College London, UK which studied a diverse list of toxins targeting various organs in the body. Each of the 107 studies (one for each toxin or physiological stressor) consisted of 30 male rats of the same strain in highly specified/controlled environments (temperature, humidity, diurnal variation, diet etc.). The collection details, applied to all studies, are depicted in Fig 2.

**Fig 3:**
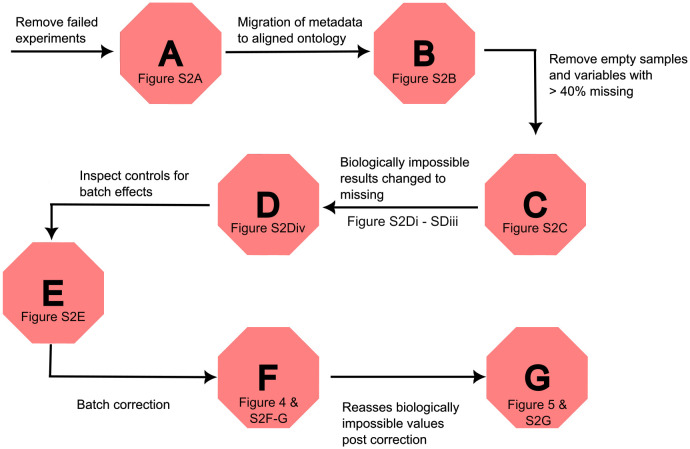
Schematic of the order of processes to curate the serum clinical chemistry parameters. Each hexagon refers to a key process that is further explained in a separate Figure.

We selected the COMET project based on several key characteristics: a large sample size (7528 serum samples with 21 clinical chemistry variables per sample), the presence of inherent structure (data generated across five independent laboratories or Companies (denoted A, B, C, D and E)), and the inevitable missing data. Like most studies, some data are absent and the inputs from multi-centre sources require careful curation and correction for batch-to-batch variation. The full process of curation is captured in Fig 3.

The first steps in Fig 3 encompass removing failed experiments (defined as use of incorrect animal species, like mice, and first attempts of studies that required repetition), harmonising labelling of metadata fields, removal of empty samples and mostly empty variables as well as biologically impossible values (e.g., negative values). Following these steps, the sample size was reduced from 7528 to 5796 and the number of clinical chemistry parameters was reduced from 21 to 12.

**Fig 4:**
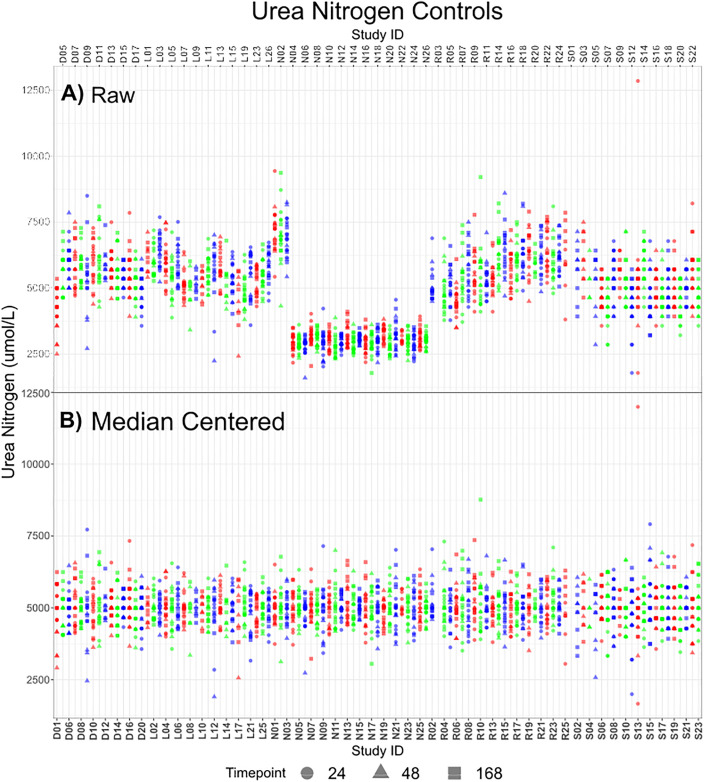
Urea Nitrogen values for control samples across all studies for A) raw data and B) Median Centered (batch corrected) data. Colours alternate for ease of visualisation.

The remaining 5796 samples were inspected for batch effects. Within a highly controlled experimental environment and the adoption of standard protocols, the control data should not present any pattern according to treatment, as this is effectively the baseline for a study. As per the experimental design (Fig 2), animals 1–10 are designated as controls that yield two samples each: a sample at 24 hours was obtained from all control rats with a second sample obtained at either 48 or 168 hours. These control samples were inspected for batch differences. The serum controls for Urea Nitrogen are displayed for each study in Fig 4A, while all remaining variables are displayed in Figure S2E in S1 File. Median centring (a shift-based correction), calculated using the control data, was applied to all data to compensate for any systematic batch differences. The median centring used the difference between the Grand Median (GM, median across all studies using the 24-hour control samples) and the Study Median (SM, the median per study for the controls inclusive of all timepoints). The corrected data are shown for Urea Nitrogen in Fig 4B. Similar results were obtained for other variable outcomes (Figure S2G in S1 File).

**Fig 5:**
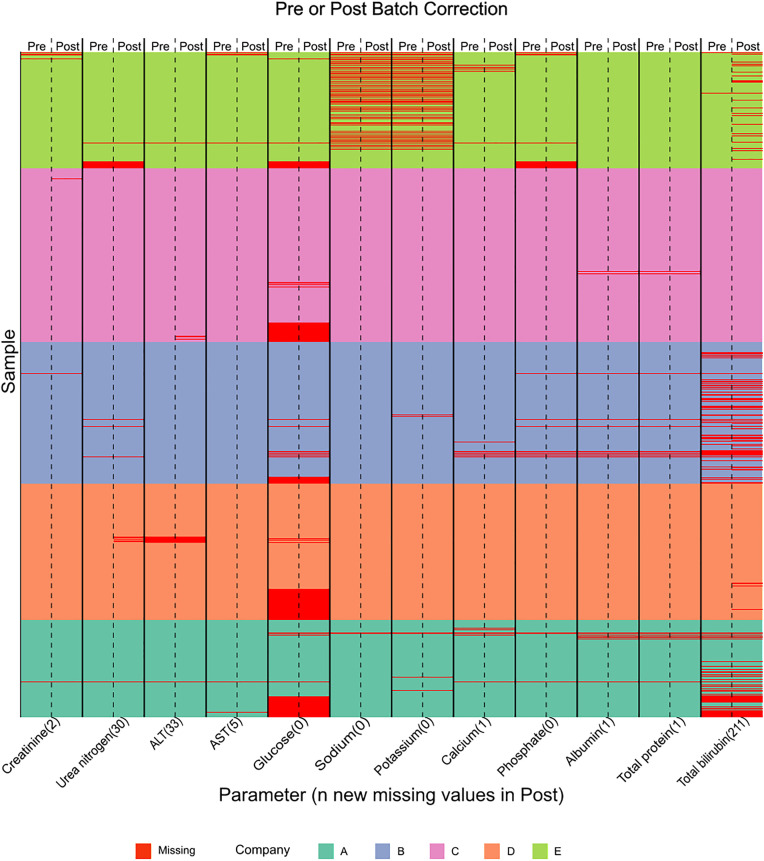
The sparsity of serum parameters pre- and post-batch correction. The y-axis depicts the sample, of which there are 5796, while the lower x-axis dictates the variable and number of values removed post batch correction. The upper x-axis denotes if it is pre- or post-batch correction.

In addition to addressing batch effects, median centring maintained biologically relevant peaks in the non-control data. The remaining variables and non-control data, along with comparison of a correction using the ratio of the SM to GM, are displayed in Figure S2Fii and S2xiii in S1 File and the batch corrected controls in Figure SG. After median centring the data were re-examined for spurious values such as negative values, which is detailed in the supplementary material. Post curation, the data remained at 5796 samples and with minimal additional sparsity as seen in Fig 5.

Of the 5796 samples 3817 complete cases were identified to create the X block (Fig 1). This large number of complete cases makes it a valuable dataset for introducing artificial missingness while having true values available for evaluation.

The knowledge about the intrinsic structure in the data mainly comes from the experimental design (Y matrix in Fig 1): company, time point, dose group (control, low dose, high dose), target of toxicity, toxin and euthanasia group. Company was selected as the C column (outcome variable) and the remaining metadata were considered the Y block (Fig 1).

All three types of missingness (MCAR, MAR and MNAR) with missing proportions of 5, 10, 20, 30 and 40%, were introduced to the 3817 complete case serum clinical sample measurements resulting in a total of 15 datasets prepared for imputation. A maximum missing proportion of 40% was chosen since this was the threshold to retain a COMET clinical chemistry parameter before curation was completed.

### Imputation

MICErf and missForest were applied to the 15 datasets with artificial missingness. The hyperparameters for number of trees was set to 10 for both MICErf and missForest to reduce bias and computational time [13,14,17]. The hyperparameter for the number of MICErf iterations was set to the recommended five for reasonable computation time [8].

As seen in Table 1, imputation was applied to: the X block (serum clinical chemistry parameters) without inclusion of metadata (X), with only C (X + C) and with the entire Y block (X + Y + C). Two different uses of the outcome variable C were evaluated: via inclusion of a C as a feature (C_Variable_) and by separate (stratified) imputation for each company (C_Strata_). The total list of combinations of variables and stratification applied are displayed in Table 1. Notably, Y_All_ did not include information about the toxin (“Toxin” field) and was completely excluded when applying missForest. This was due to the maximum number of levels per factor being limited to 53 when using missForest, while there are 107 different toxins and stressors. The 11 imputation strategies described in Table 1 were applied to the 15 datasets, generated from five different levels of missingness (M = 5, 10, 20, 30 and 40%) and three different missingness types (MT = MAR, MCAR and MNAR) for the 3817 complete cases. These 15 datasets were repeated for two additional sample sizes (N = 50 and 500). Imputation for each dataset was repeated 20 times with metrics from Table S2 in S1 File. Each metric had its mean and standard deviation from the iterations recorded. Results for the reduced sample sizes can be found in the supplementary material. All analysis was performed using R (version 4.2.2), with the software packages *mice* 3.16.0 [33] and *missForest* 1.5 [34] used for imputation. Missingness was generated using the package *missMethods* 0.4.0. As MICE produces multiple imputed data sets, the package *sjmisc* 2.8.9 [35] was used to merge them into a single result.

**Table 1:** Combinations of variable inclusion and stratification approaches where X is the clinical chemistry dataset that is missing values. C is the outcome variable, with C_Strata_ representing separate imputation per group defined in the chosen variable and C_Variable_ is including the outcome as a variable. Y_All_ is the remaining metadata not used for stratification. Y_All + Toxin_ is the same as Y_All_ but with Toxin metadata now included.

MICErf	MissForest
X	X
X + C_Strata_	X + C_Strata_
X + C_Variable_	X + C_Variable_
X + Y_All _+ C_Strata_	X + Y_All _+ C_Strata_
X + Y_All _+ C_Variable_	X + Y_All _+ C_Variable_
X + Y_All+Toxins _+ C_Variable_	

### Performance metrics

Best practice guidelines for imputation are still evolving and the validity of the methods should be checked using multiple performance metrics [14,36]. The resulting complete-by-imputation datasets were assessed using internal performance metrics such as Normalised Root Mean Square Error (NRMSE) and Mean Absolute Error (MAE). Additionally, external performance metrics for Bias (using existing relationships) and distribution analysis were applied. Full details are provided in Table S2 in S1 File.

Random Forest (RF) Machine Learning (ML) imputation methods were selected for evaluation. Their accuracy and maintenance of predictive ability (robustness) currently outperform traditional [14,17,37,38] and Deep Learning (DL) [12,37] imputation methods. ML-based imputation is more suitable for high-dimensional data with complex inherent structure and collinear variables [39] and the utilisation of RF does not require the same detailed and accurate specifications in the parameters or pre-imputation transformation of the data to cope with variable interactions, non-linearities and non-normal distributions as non-RF methods such as parametric MICE [13–20,40]. RF based methods also implement some degree of automated variable selection as the algorithm can choose not to incorporate or extremely minimise the impact of available features [37,40,41]. For these reasons MICE based on RF (MICErf) [33] and missForest, in combination with stratification options, were given a comparative assessment of robustness, maintenance and accuracy using the metrics in Table S2 in S1 File using the COMET data. To the best of our knowledge, no prior publications have addressed these considerations.

## Results

### Stratification

**Fig 6:**
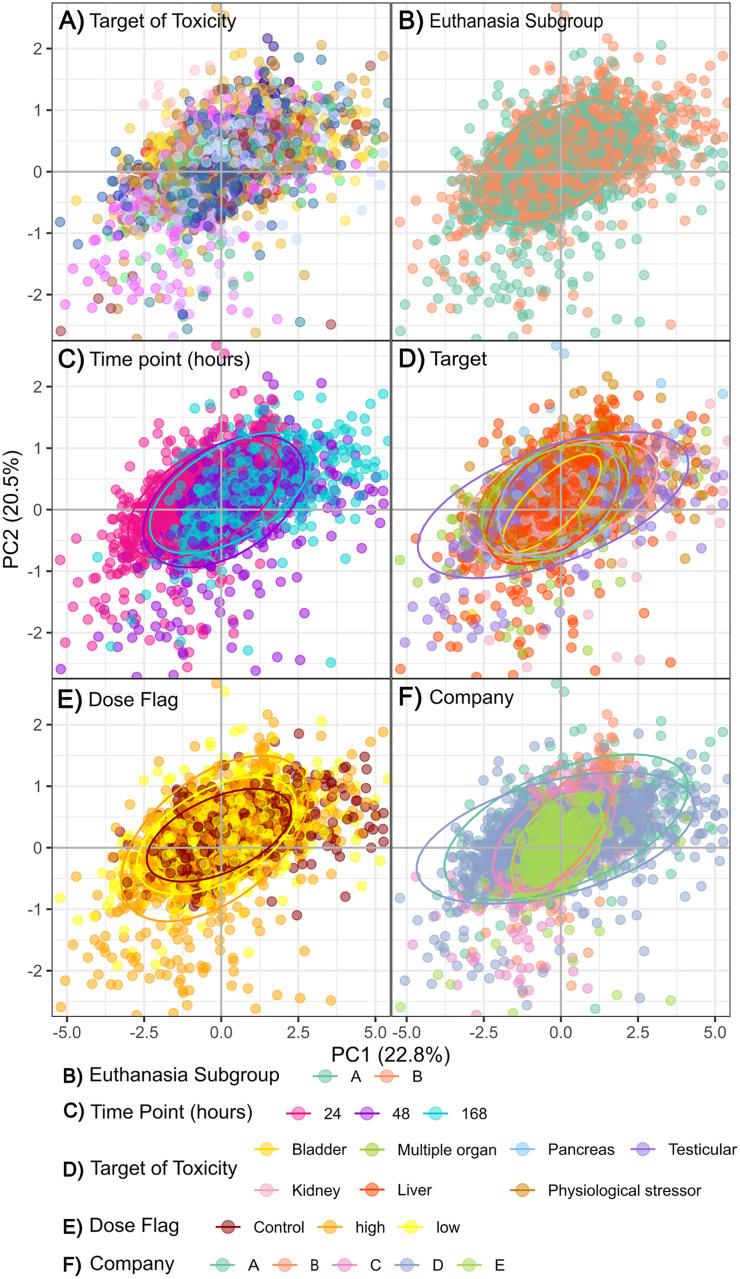
Zoomed-in PCA scores plot of the 3817 complete cases for serum clinical chemistry parameters. The range was restricted to ± 5 for visualisation purposes, highlighting groupings based on the metadata variables **A)** ‘Toxin’, **B)** ‘Euthanasia Subgroup’, **C)** ‘Time Point (hours)’, **D)** ‘Target of Toxicity’, **E)** ‘Dose Flag’ and **F)** ‘Company’ for the first two principal components.

Unsupervised multivariate analysis using Principal Component Analysis (PCA) of the 3817 complete case serum clinical measurements was conducted. The scores of the first two principal components are displayed in Fig 6 and coloured according to the serum variables. Fig 6 has been restricted to scores of ± 5 for visualisation purposes, capturing 90% of the data. While the clusters of samples by Company are not distinctly separated in Fig 6F, their presence, even with overlap, indicates grouping. Company was designated the outcome variable (C) for this study because any influence it exerts is undesirable and would require correction. In contrast the variables time point, dose group, target of toxicity, toxin and euthanasia group are pertinent to the study’s purpose and were not considered as confounders. Hence, these remaining metadata variables constitute the remainder of our Y block.

### Imputation method

**Fig 7:**
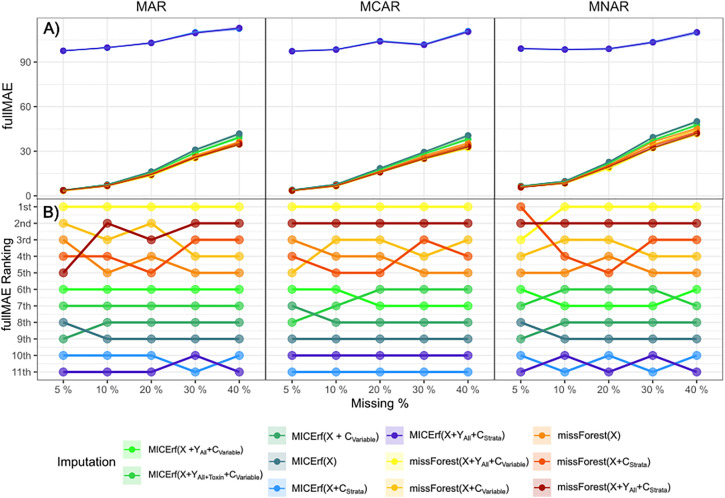
Internal performance metric full MAE for varying proportions of missing data for all three types of missingness (MT = MCAR, MAR and MNAR) using imputation methods missForest and MICErf. **A)** the full MAE values where the solid line and points represent the mean values averaged over 20 iterations, while the shadow denotes the confidence interval. **B)** The Bump Chart for method performance ranked 1^st^ (best) to 11^th^ (worst).

A total of 11 different imputation methods/strategies described in Table 1 were tested on the fifteen datasets with various artificial missingness (MT = MAR, MCAR and MNAR at M = 5, 10, 20, 30 and 40%). Each imputation method was iterated 20 times with the mean and standard deviation of the performance metrics recorded. The internal metrics, representing accuracy and precision all followed the same trend as shown in Fig 7 for the full MAE. The NRMSE and partial MAE can be found in the supplementary material.

As has been observed with other types of data [17,38], the missForest algorithm consistently, if only slightly, outperformed MICErf in terms of accuracy and precision by having the lowest internal metrics under every type of missingness. The combinations of stratification and variable inclusion described in Table 1 had minimal impact when applying missForest. The full MAE was tightly clustered for the missForest points in Fig 7A and maintained the top five ranks in Fig 7B. However, stratification via separate imputation per company (C_strata_) had a pronounced effect on MICErf, consistently being the lowest ranked and significantly deviating from all other methods.

As mentioned, the inclusion of the Y block and inclusion or choice of C_Strata_ or C_Variable_ caused minimal disparity between the missForest results. Results for smaller sample sizes, as seen in Figures S4–S6 in S1 File, still designated missForest as the more robust out of the two methods. Of note is the fact that stratified MICErf was unable to complete any imputation for a sample size of 50 and was unsuccessful at imputing one to two variables for a sample size of 500 for various missingness types and proportions.

**Fig 8:**
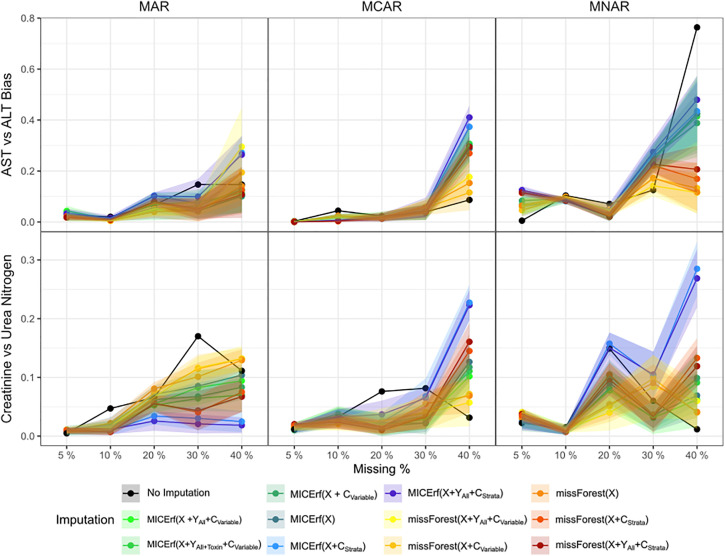
External performance metric of imputation bias for varying proportions of missing data for all three types of missingness (MCAR, MAR and MNAR) using imputation methods missForest and MICErf. The bias is measured with respect to the linear relationship between AST versus ALT and Creatinine versus Urea Nitrogen. Mean values after 20 iterations are represented by the solid line with points and standard deviation by the shadow.

Within the twelve serum clinical parameters, “AST” and “ALT”, alongside “Creatinine” and “Urea Nitrogen” variables, are biologically connected and are known to hold linear relationships. These relationships had higher correlations than other variable pairs, with ALT versus AST having an R^2^ of 0.86 and Creatinine versus Urea Nitrogen having an R^2^ of 0.72 (seen in Figure S7 in S1 File). The complete list of correlations can be found in Figure S8 in S1 File. Using this prior knowledge, Fig 8 illustrates the degree of bias introduced by each imputation method in comparison to not imputing as described in Table S1 in S1 File.

Optimal methods in terms of bias oscillate between not imputing, missForest and MICErf depending on the type and proportion of missingness. Similar, the internal metric in Fig 7, C_Strata_ often has a negative effect on MICErf. Although missForest does not always outperform MICErf, the majority of the unstratified missForest results either outperform or are on par with the MICErf results. The comparison between two sample sizes (N = 500 and 50) shown in Figures S9 and S10 in S1 File demonstrates that, majority of the time, missForest still outperforms MICErf, further supporting the use of missForest when choosing to impute.

The distributions pre- and post-imputation for 40% missing values, found in the supplementary material (Figure S11 in S1 File), did not raise concerns, as they did not display any major deviations in distribution from the original (CCA) data distribution**.** We selected 40% as a worst-case scenario.

## Discussion

With no clear distinction in preferable performance in the distributions in Figure S11 in S1 File, and examination of the internal and external performance metrics displayed in Figs 7 and 8, missForest was identified as the most suitable imputation method for all missingness types and up to 40% of missing data, confirming earlier reports on different types of data [17,38]. If choosing to impute with MICErf, stratification should be avoided due to its large negative effect on the internal metrics displayed in Fig 7. This holds true for our largest sample size of 3817 for all combinations of features and stratification methods. While missForest remains the optimal imputation method for the smaller sample sizes, unstratified MICErf is still a close competitor (Figures S3 and S4 in S1 File).

The imputation and data processing strategy presented here allowed the alignment and removal of systematic batch differences between studies. In regard to chemical pathology data, this is of value for biological interpretation since clinical chemistry variables, particularly electrolytes and enzymes are known to be prone to variation [42,43] and therefore, the ability to co-analyse data from studies adds statistical power to establishing expected ranges for these parameters. However, the evaluation of the imputation pipeline presented here has a broader relevance to large-scale biological and chemical datasets. The fact that missForest delivers similar results with both stratification and variable inclusion makes it a suitable candidate for large databanks where data are imputed sequentially, as more datasets are added.

It is important to note that our comparisons do not encompass all possible data transformations prior to imputation, nor do they consider fine-tuning hyperparameters, such as the number of trees. Optimum hyperparameters are intrinsically dependent of the dataset of interest and lies beyond the scope of our current study but warrants further investigation. We recognize that by varying hyperparameters, the relative performance of MICErf and missForest may improve substantially. Nevertheless, we illustrate the effectiveness of missForest, which operates well and robustly with minimal user intervention, aligning well with our research objectives. Our recommendation of missForest is primarily based on its robustness to user error and its consistent performance across missingness types and proportion and sample size.

## Conclusion

The results of the simulation of varying proportions and types of missingness in an exemplary large dataset support missForest as an ideal and robust imputation method. We recommend missForest as the most method robust to user error. And, if choosing to impute by MICErf, we recommend to avoid stratification due to its large impact on performance at all sample sizes. Further examination of the effect of fine tuning hyperparameters on relative performance of missForest and MICErf is required, and our recommendations is only to apply the approach in cases for up to 40% missingness. These results highlight, once more, the necessity to store the raw data (prior to any processing) so that imputation or any further correction can be applied on the merged data if necessary.

## Supporting information

S1 File Supplementary figures Fig S1 to Fig S11. (DOCX)
