# Supplementary material for: Evaluation of imputation strategies for multi-centre studies: Application to a large clinical pathology dataset
Source: PLoS One. 2025 Nov 20;20(11):e0335852. doi: 10.1371/journal.pone.0335852 (PMC12633936; doi:10.1371/journal.pone.0335852)
Supplement: S1 File — (DOCX) [file pone.0335852.s001.docx]

# **Supplementary Material**

# **Evaluation of imputation strategies for multi-centre studies: Application to a large clinical pathology dataset.**

Lucy Grigoroff^1†^, Reika Masuda^1^, John Lindon^2^, Janonna Kadyrov^1^, Jeremy K. Nicholson^1,2^, Elaine Holmes^1,3^, Julien Wist^1,3,4†^.

*^1^Australian National Phenome Centre, and Centre for Computational and Systems Medicine, Health Futures Institute, Murdoch University, Harry Perkins Building, Perth, WA6150, Australia.*

*^2^Institute of Global Health Innovation, Faculty of Medicine, Imperial College London, Level 1, Faculty Building, South Kensington Campus, London, SW7 2NA, UK.*

*^3^Department of Metabolism, Digestion and Reproduction, Faculty of Medicine, Imperial College London, Sir Alexander Fleming Building, South Kensington, London SW7 2AZ, UK.*

*^4^Chemistry Department, Universidad del Valle, 76001, Cali, Colombia.*

^†^Corresponding author: Lucy Grigoroff – [Lucygrigoro@gmail.com](mailto:Lucygrigoro@gmail.com)

^†^Corresponding author: Julien Wist – Julien.wist@murdoch.edu.au

**Table of Content**

[**Supplementary Material** 1](#_Toc178160794)

[**Evaluation of imputation strategies for multi-centre studies: Application to a large clinical pathology dataset.** 1](#_Toc178160795)

[Normalised Root Mean Squared Error (NRMSE) 3](#_Toc178160796)

[Mean Absolute Error (MAE) 3](#_Toc178160797)

[Bias 3](#_Toc178160798)

[Data Processing 4](#_Toc178160799)

[Figure S1 4](#_Toc178160800)

[Figure S2A. 5](#_Toc178160801)

[Figure S2B. 6](#_Toc178160802)

[Figure S2C. 7](#_Toc178160803)

[Figure S2Di 8](#_Toc178160804)

[Figure S2Dii. 9](#_Toc178160805)

[Figure S2Diii 10](#_Toc178160806)

[Figure S2Div. 11](#_Toc178160807)

[Figure S2E. 12](#_Toc178160808)

[Figure S2Fi. 13](#_Toc178160809)

[Figure S2Fii. 14](#_Toc178160810)

[Figure S2Fiii. 15](#_Toc178160811)

[Figure S2Fiiv. 16](#_Toc178160812)

[Figure S2Fv. 17](#_Toc178160813)

[Figure S2Fvi. 18](#_Toc178160814)

[Figure S2Fvii. 19](#_Toc178160815)

[Figure S2Fviii. 20](#_Toc178160816)

[Figure S2Fix. 21](#_Toc178160817)

[Figure S2Fx. 22](#_Toc178160818)

[Figure S2Fxi. 23](#_Toc178160819)

[Figure S2Fxii. 24](#_Toc178160820)

[Figure S2Fxiii. 25](#_Toc178160821)

[Figure S2G. 26](#_Toc178160822)

[Figure S2H. 27](#_Toc178160823)

[Table S1. 27](#_Toc178160824)

[Table S2 28](#_Toc178160825)

[Figure S3. 29](#_Toc178160826)

[Figure S4. 30](#_Toc178160827)

[Figure S5. 31](#_Toc178160828)

[Figure S6. 32](#_Toc178160829)

[Figure S7. 33](#_Toc178160830)

[Figure S8. 34](#_Toc178160831)

[Figure S9. 35](#_Toc178160832)

[Figure S10. 36](#_Toc178160833)

[Figure S11. 41](#_Toc178160834)

### Normalised Root Mean Squared Error (NRMSE)

$$NRMSE =\frac{\sqrt{\frac{\sum_{i=1}^{n} {({x_{oi}-x_{pi}})^{2}}}{n}}}{\underline{x}_{o}}$$

where $X_{0}$ is the actual value, $X_{p}$ is its imputed value, $\underline{X_{0}}$ is the mean of the observed values and *n* is the total number of observations.

### Mean Absolute Error (MAE)

$MAE= \frac{\sum_{i=1}^{n} \left| x_{oi}-x_{pi} \right|}{n}$

The MAE was calculated using both partial datasets (only the imputed and corresponding original values, partialMAE) and complete datasets (based on the entire data, fullMAE).

### Bias

$$Bias = \frac{\left| {Slope}_{original}-{Slope}_{changed} \right|}{{Slope}_{original}}$$

Where Slope_original_ is the slope of the total 3,817 cases before artificially created missingness and Slope_changed_ is the slope of either only the CCA post artificial missingness or the resulting imputed dataset.

### Data Processing

Prior to selecting complete cases for this study, some cleaning and transformation of the data took place, as seen in **Figure 3** of the main text.


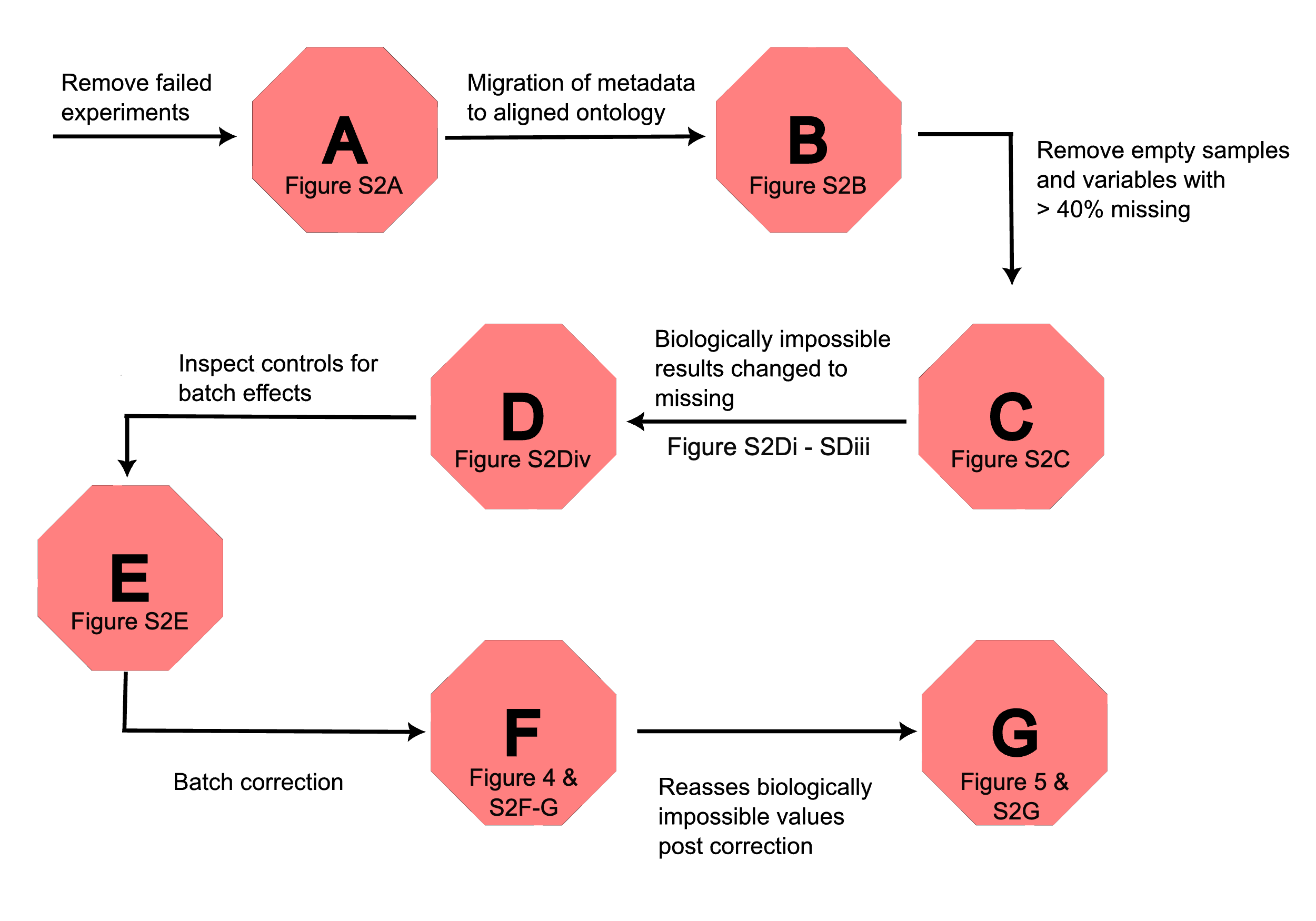


### Figure S1

Schematic of the order of processes to curate the serum clinical chemistry parameters. Each hexagon refers to a more detailed image.


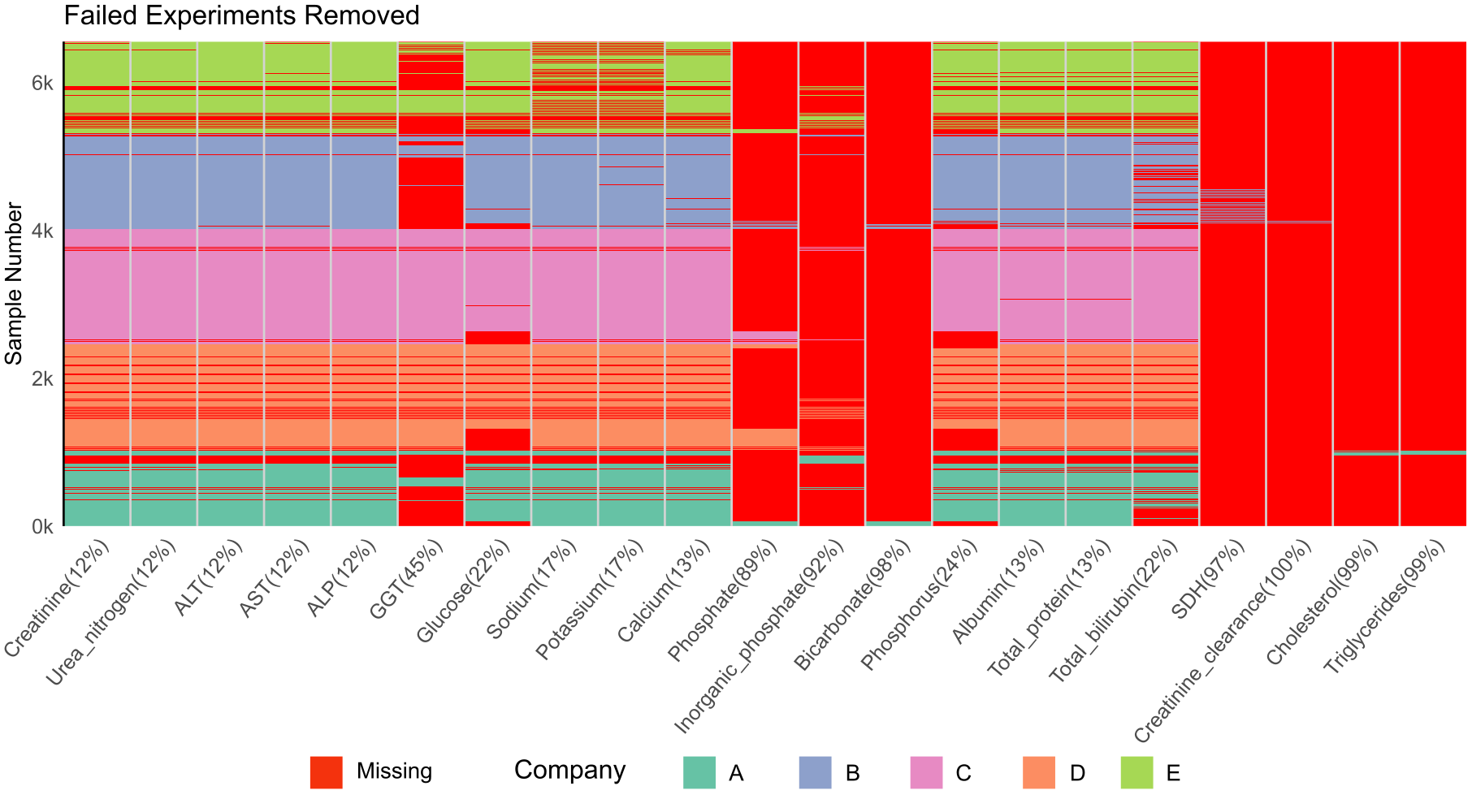


### Figure S2A.

The sparsity of serum parameters once failed experiments are removed. The y axis depicts the sample number while the x axis dictates the variable and percentage missing as variable (%).

It was noted that inclusion of phosphorus was a notation error that should have been phosphate. All present values for phosphorus were missing from phosphate, further confirming this as a notation error by the participating companies. The values were appropriately transferred to phosphate as seen in **Figure S2B.**


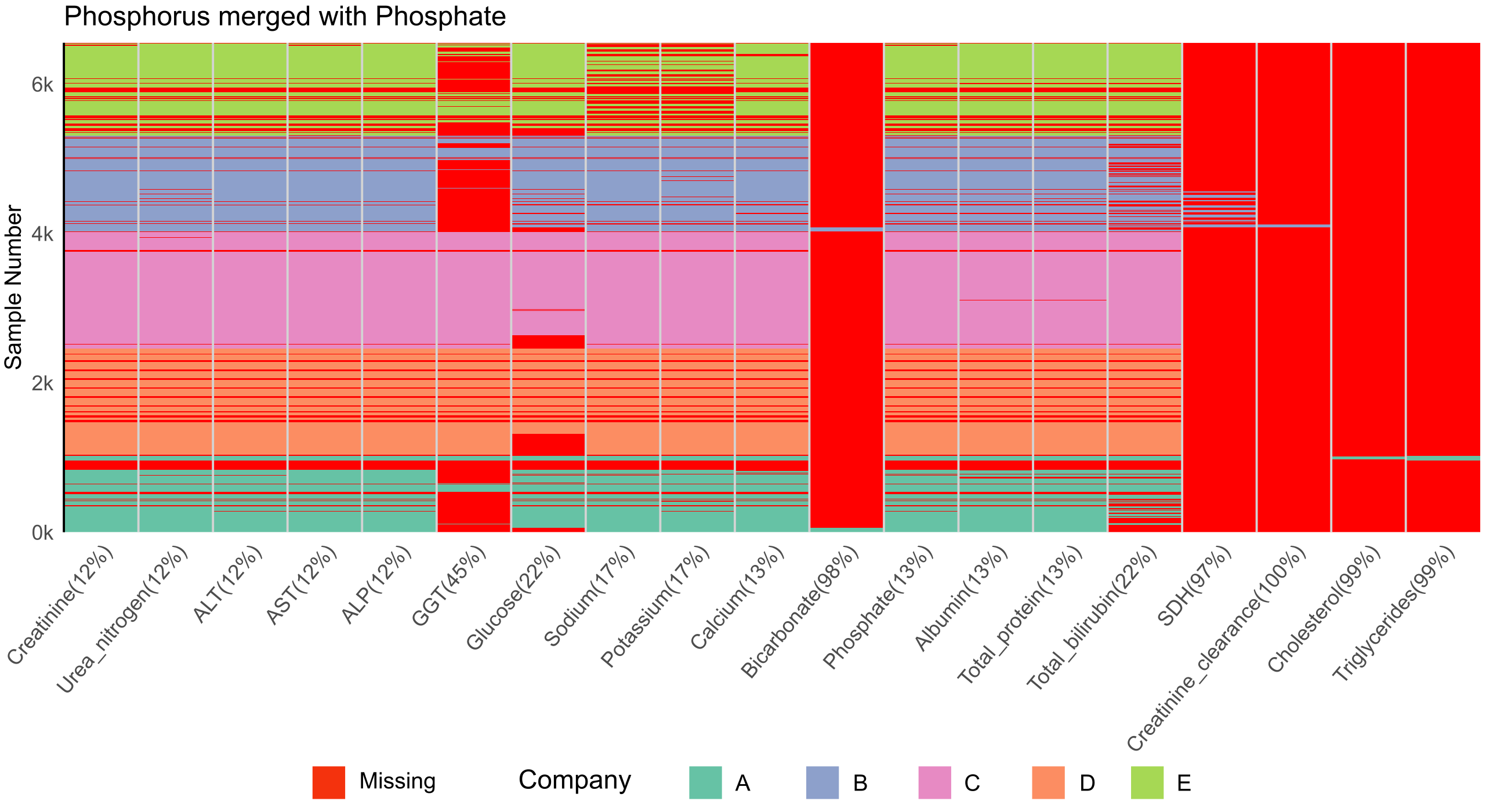


### Figure S2B.

The sparsity of serum parameters once phosphorus values were relabelled as phosphate. The y axis depicts the sample number while the x axis dictates the variable and percentage missing as variable (%).

Post relabelling phosphorus to phosphate, all empty samples were removed. Post deletion, if the variables had more than 40% still missing, they were also removed. The resulting sparsity is displayed in **Figure S2C**.


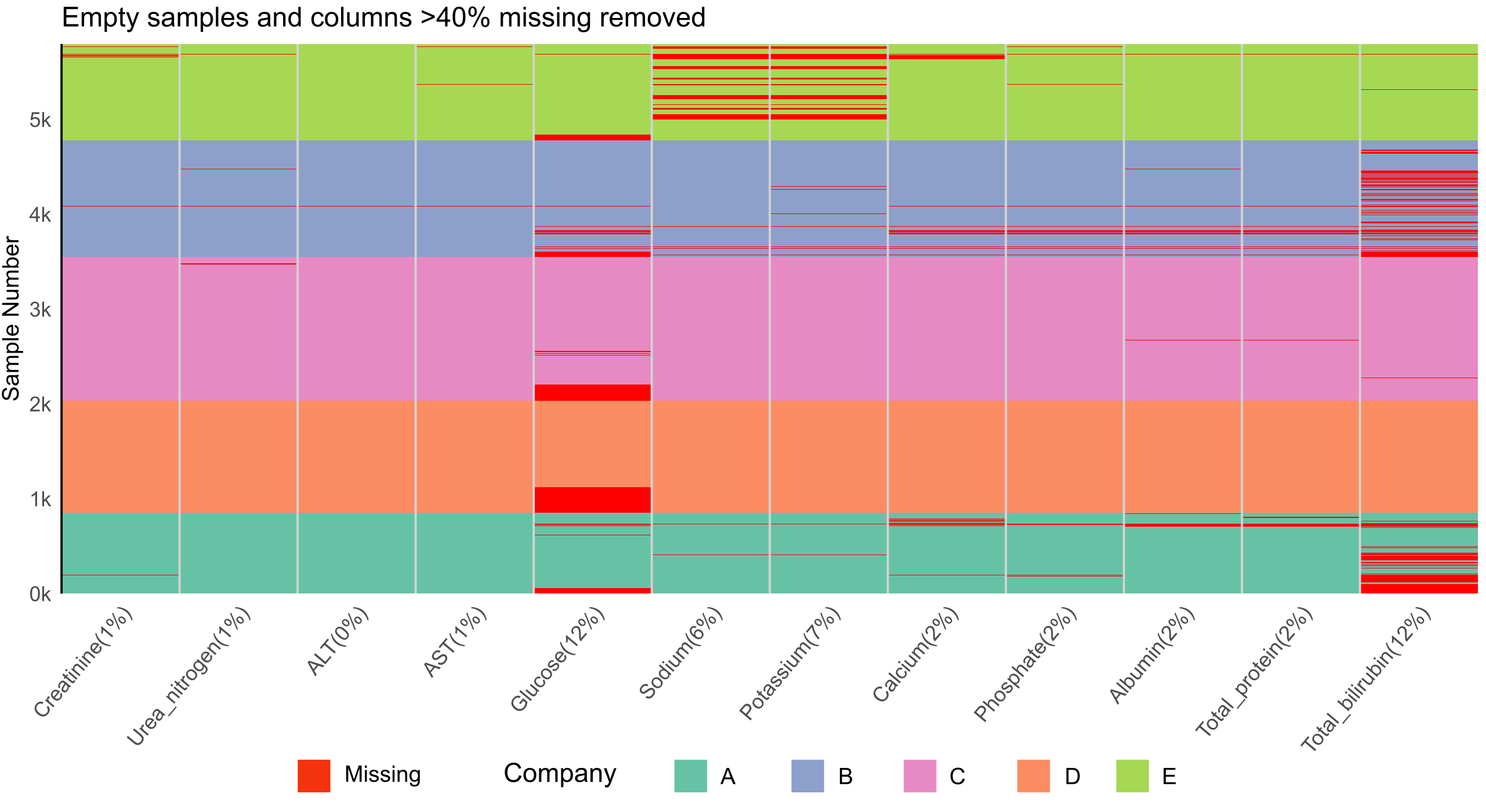


### Figure S2C.

The sparsity of serum parameters with empty samples and variables more than 40% empty removed. The y axis depicts the sample number while the x axis dictates the variable and percentage missing as variable (%).

The data was examined for biologically impossible values post sample and variable deletion. This was based on the advice from XX Elaine Holmes. Any negative values were automatically changed to missing. Other than negative values, restrictions of Urea nitrogen < 1000 umol/L, Sodium < 10 mmol/L, Calcium >70 mmol/L and phosphate < 0.5 mmol/L were applied. All applied thresholds are depicted in **Figure S2Di-iii**. Studies S01 for phosphate, and D05 and D06 for Glucose were changed to missing due to the elevated values for the entire study, including the controls. These are annotated in **Figure S2Dii.-iii** and the new sparsity is depicted in **Figure S2Div.**


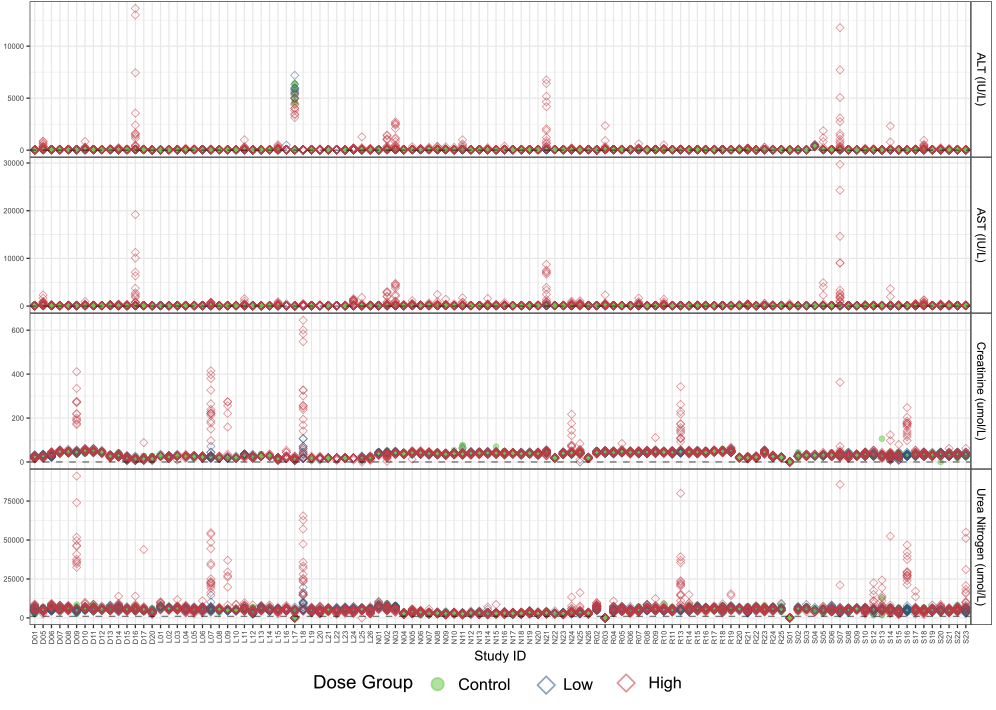


### Figure S2Di

Study versus variables ALT, AST, creatinine and urea nitrogen coloured by dose group. The Dashed grey lines represent thresholds applied for which below the values are deemed biologically impossible and changed to missing.


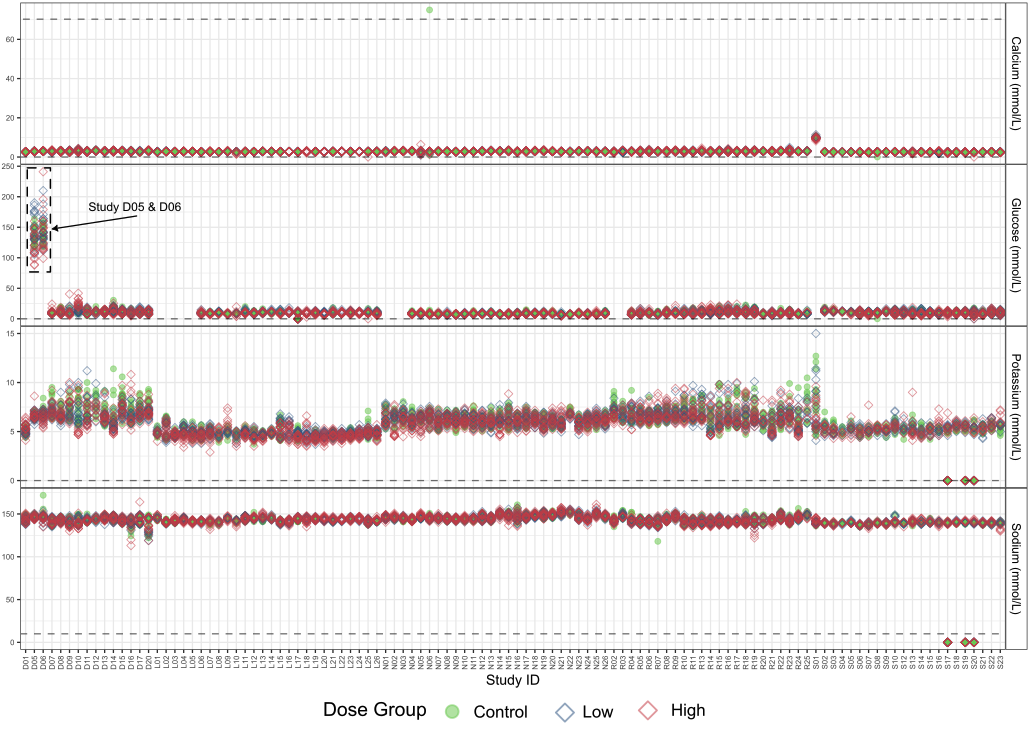


### Figure S2Dii.

Study versus variables calcium, glucose, sodium and potassium coloured by dose group. The Dashed grey lines represent thresholds applied for which below the values are deemed biologically impossible and changed to missing. Study D05 and D06 are annotated since they were also changed to missing.


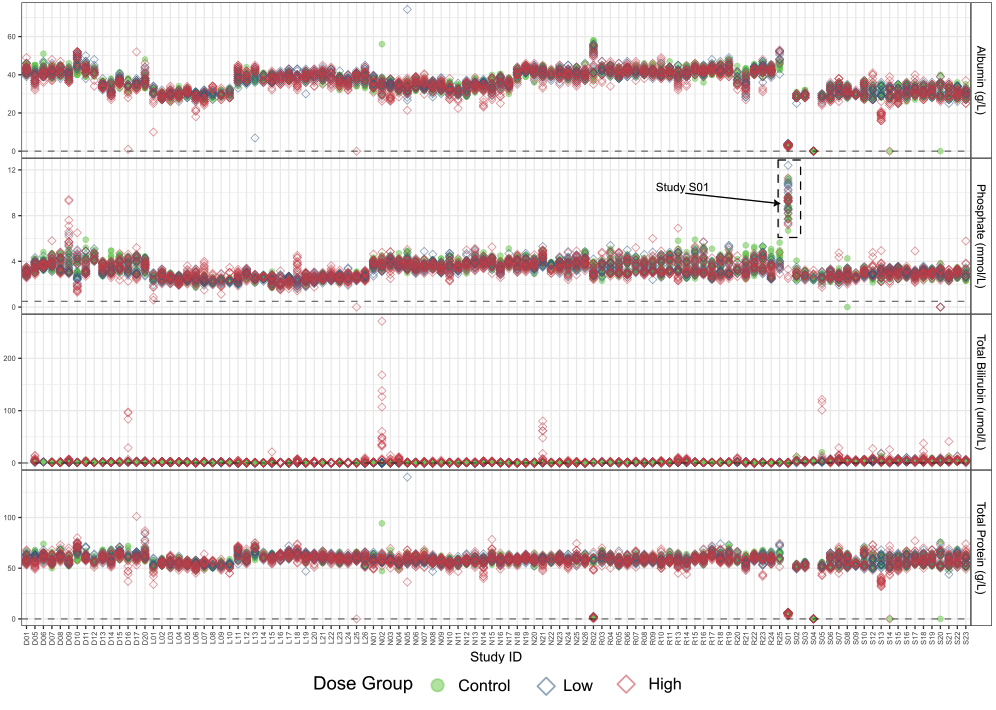


### Figure S2Diii

Study versus variables albumin, phosphate, total protein, total bilirubin coloured by dose group. The Dashed grey lines represent thresholds applied for which below the values are deemed biologically impossible and changed to missing. Study S01 is annotated for phosphate since they were also changed to missing.


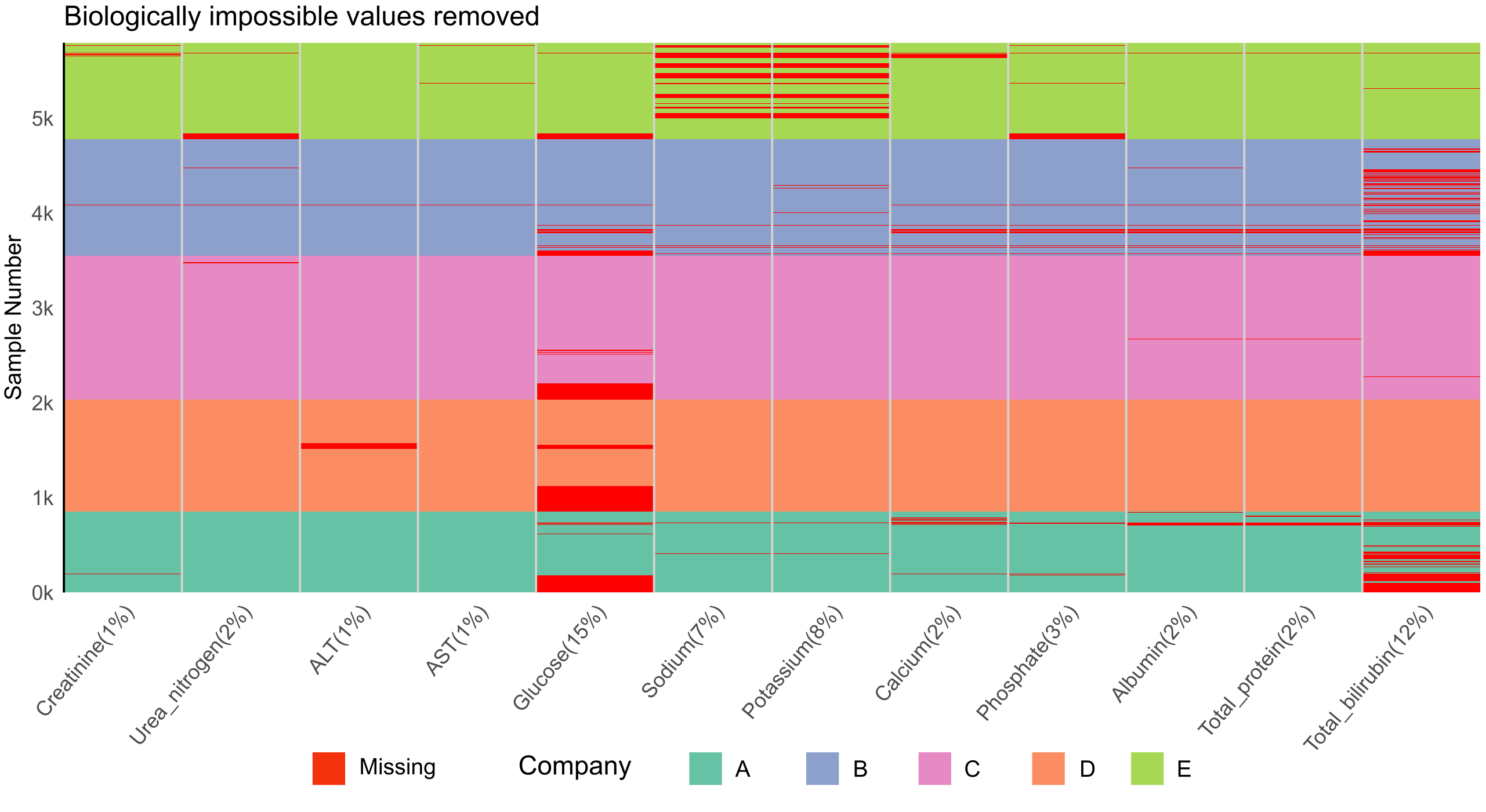


### Figure S2Div.

The sparsity of serum parameters with biologically impossible values removed. The y axis depicts the sample number while the x axis dictates the variable and percentage missing as variable (%).

Once the biologically impossible values were removed, the data was inspected for batch effects. The controls alone were inspected for batch corrections. With such a highly controlled experimental environment, the controls are not expected to present with patterns. The serum controls are displayed by study in **Figure S2E**.


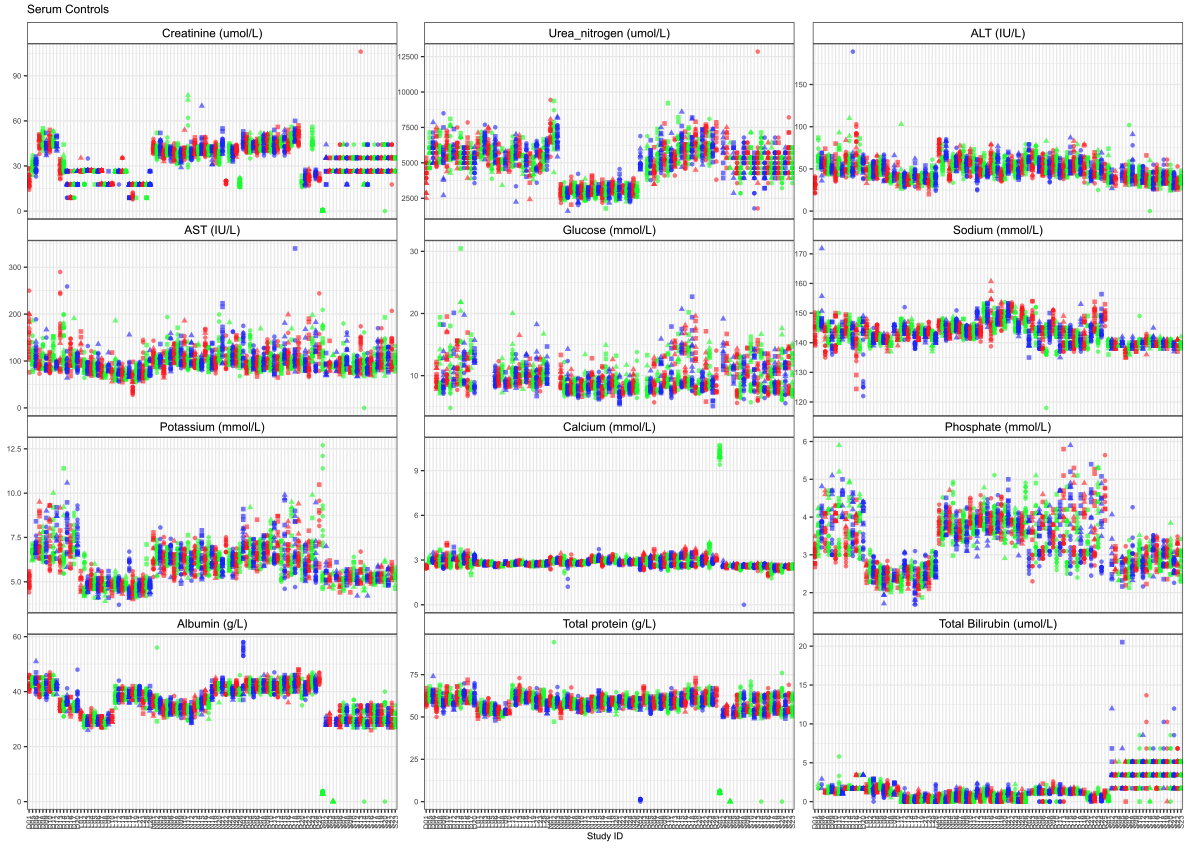


### Figure S2E.

Variables versus study for control data only. Studies alternate colour for visualisation purposes. Batch effects are noticeably present for most variables.

As seen in **Figure S2E.**, most variables suffer from a degree of batch effects. Shift and ratio-based corrections were considered using the control data.

C_Shift_ = SM - GM

X_Corrected_ = X - C_Shift_

C_Ratio_ = GM/SM

X_Corrected_ = X * C_Ratio_

Where X_Corrected_ is the value post correction, X is the raw value, SM and GM are the study and grand median, respectively and C_Shift_ and C_Ratio_ are the shift and ratio-based correction factors. While the corrections are based on the controls, they are applied to all data. For each variable, the GM was the median of the 24-hour time point from all studies combined, while the SM was the median of individual studies controls inclusive of all time points. For both corrections, where no study median is available, the average SM for other studies from the same company is used.

Both corrections were applied and evaluated based on their ability to reduce the total variance of the controls (**Figure S2Fi.**), minimise batch effects, and preserve biologically relevant peaks in the complete data (**Figure S2Fii. - Figure S2Fxiii.**)


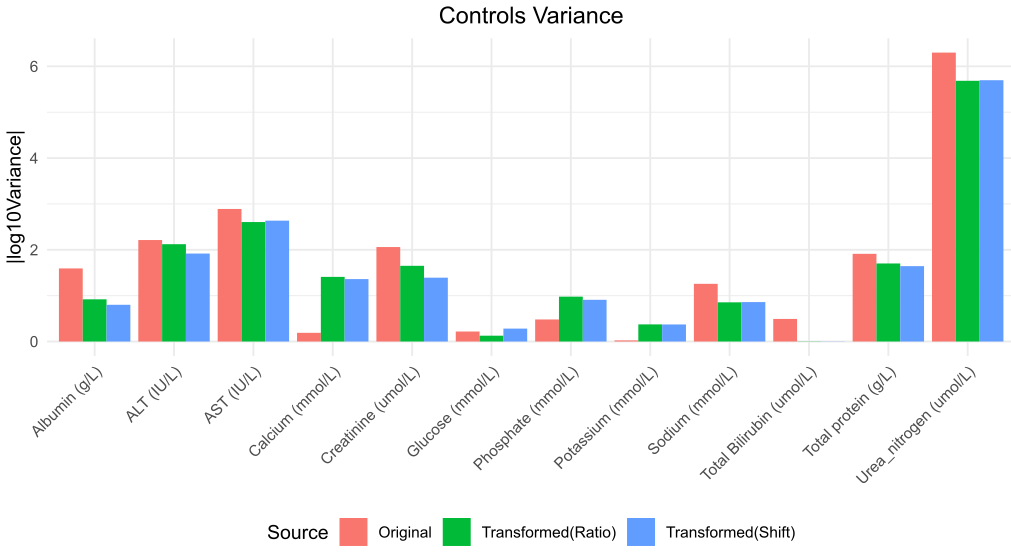


### Figure S2Fi.

The variance of the controls before (original) and after (transformed) correction.


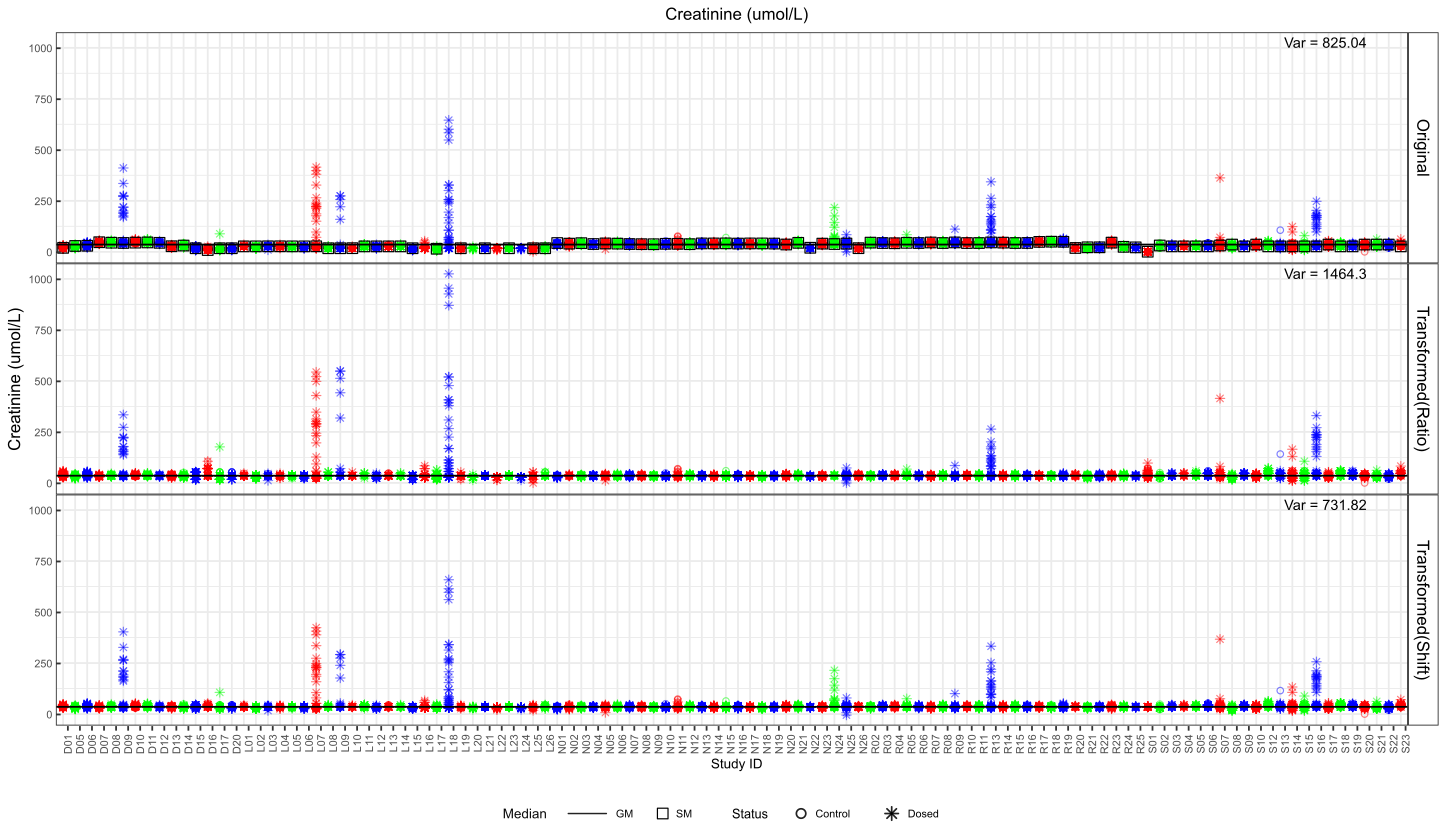


### Figure S2Fii.

Creatinine before (Original) and after batch corrections (Ratio and Shift). The total variance is displayed in the top right corner of each plot facet. Studies alternate colour for visualisation purposes.

_
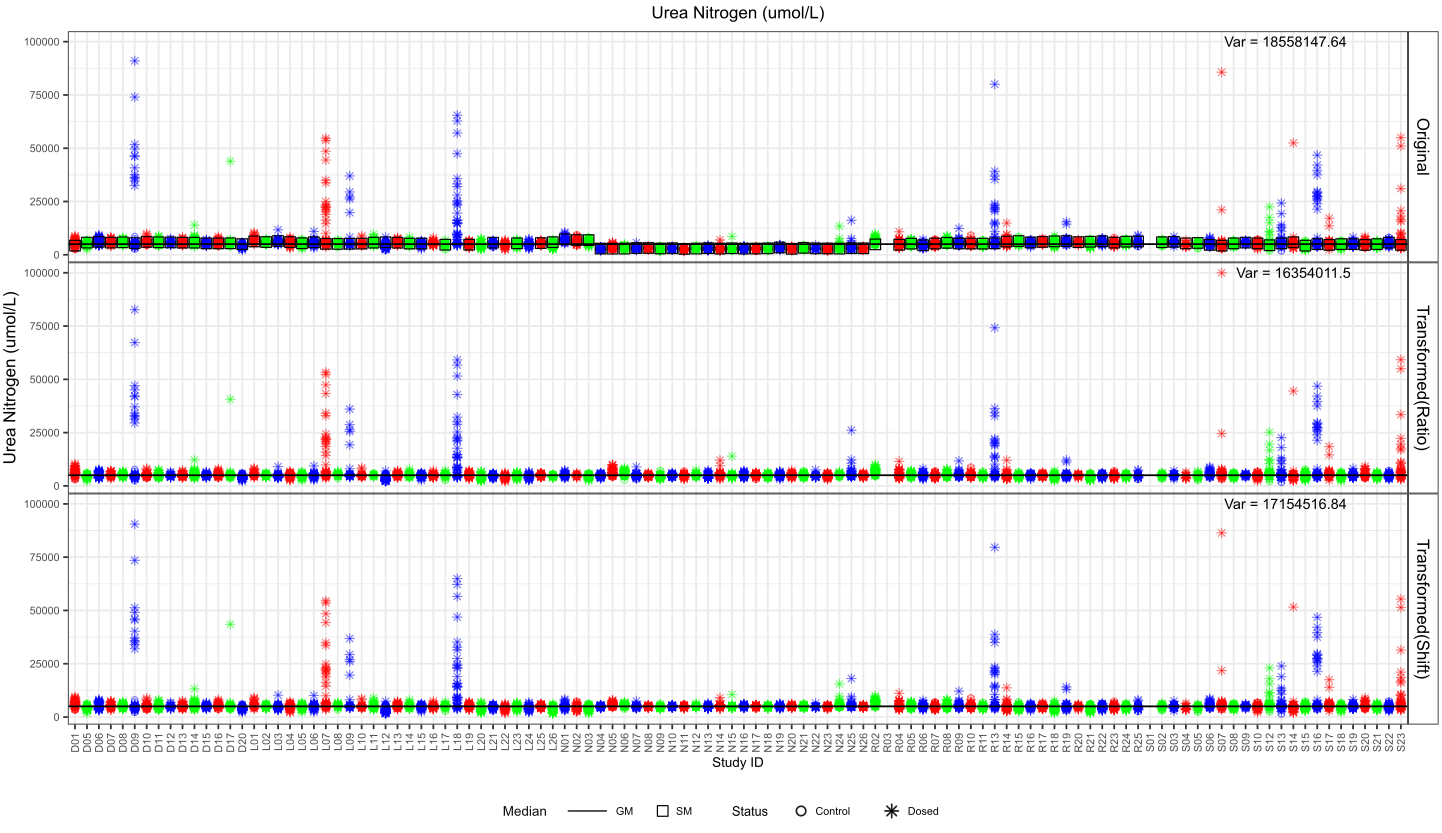
_

### Figure S2Fiii.

Urea Nitrogen before (Original) and after batch corrections (Ratio and Shift). The total variance is displayed in the top right corner of each plot facet. Studies alternate colour for visualisation purposes.


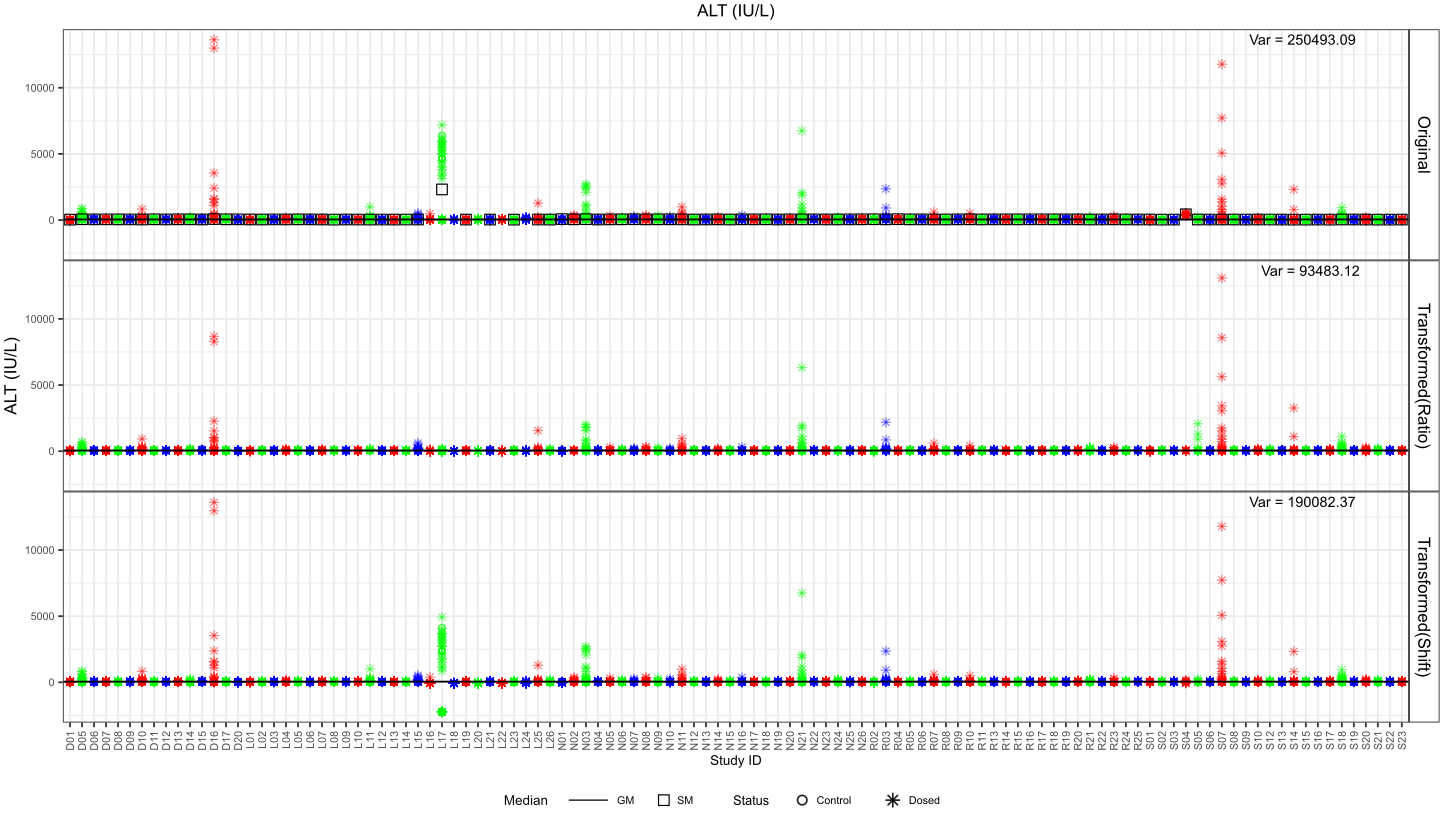


### Figure S2Fiiv.

ALT before (Original) and after batch corrections (Ratio and Shift). The total variance is displayed in the top right corner of each plot facet. Studies alternate colour for visualisation purposes.


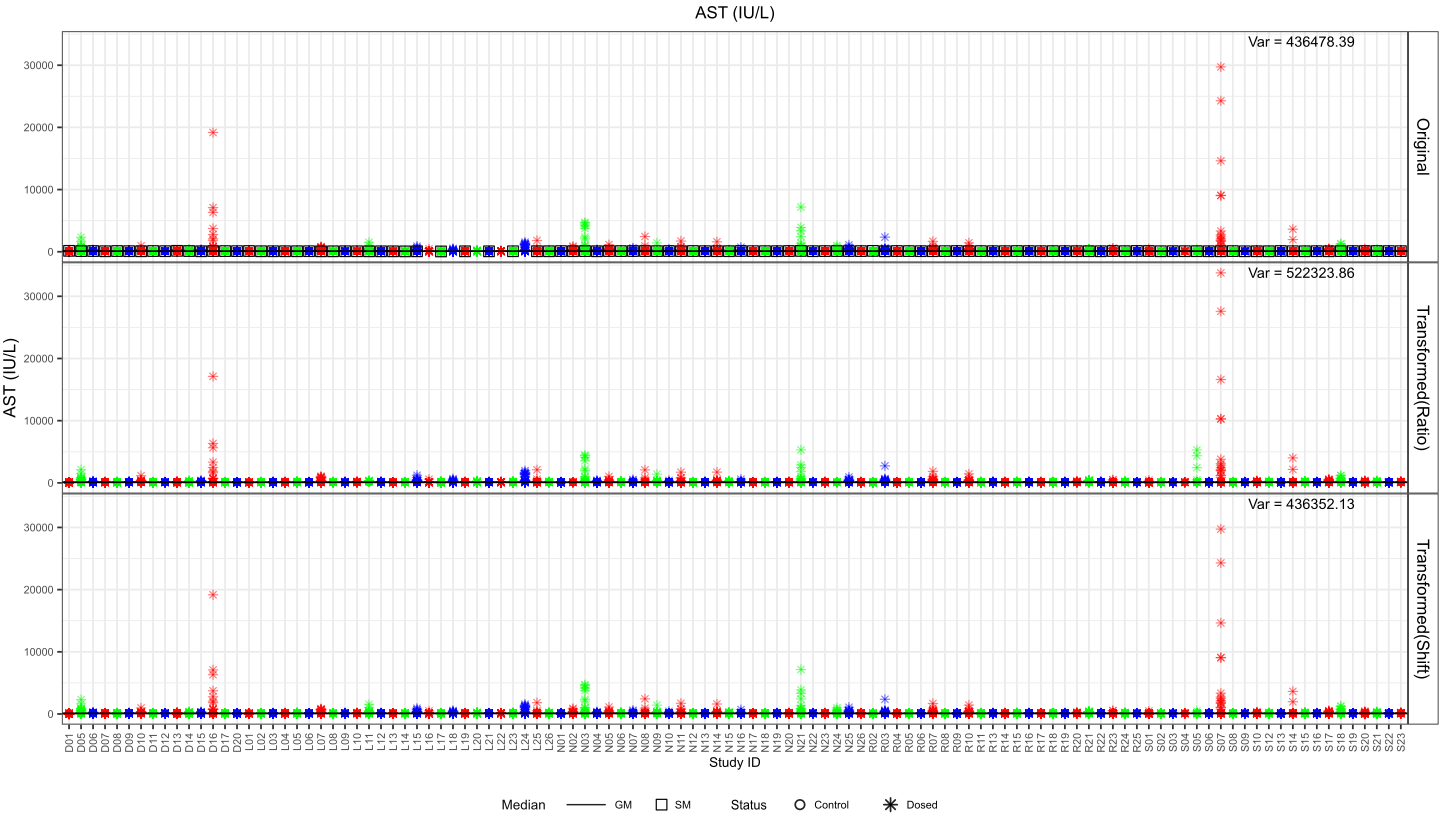


### Figure S2Fv.

AST before (Original) and after batch corrections (Ratio and Shift). The total variance is displayed in the top right corner of each plot facet. Studies alternate colour for visualisation purposes.


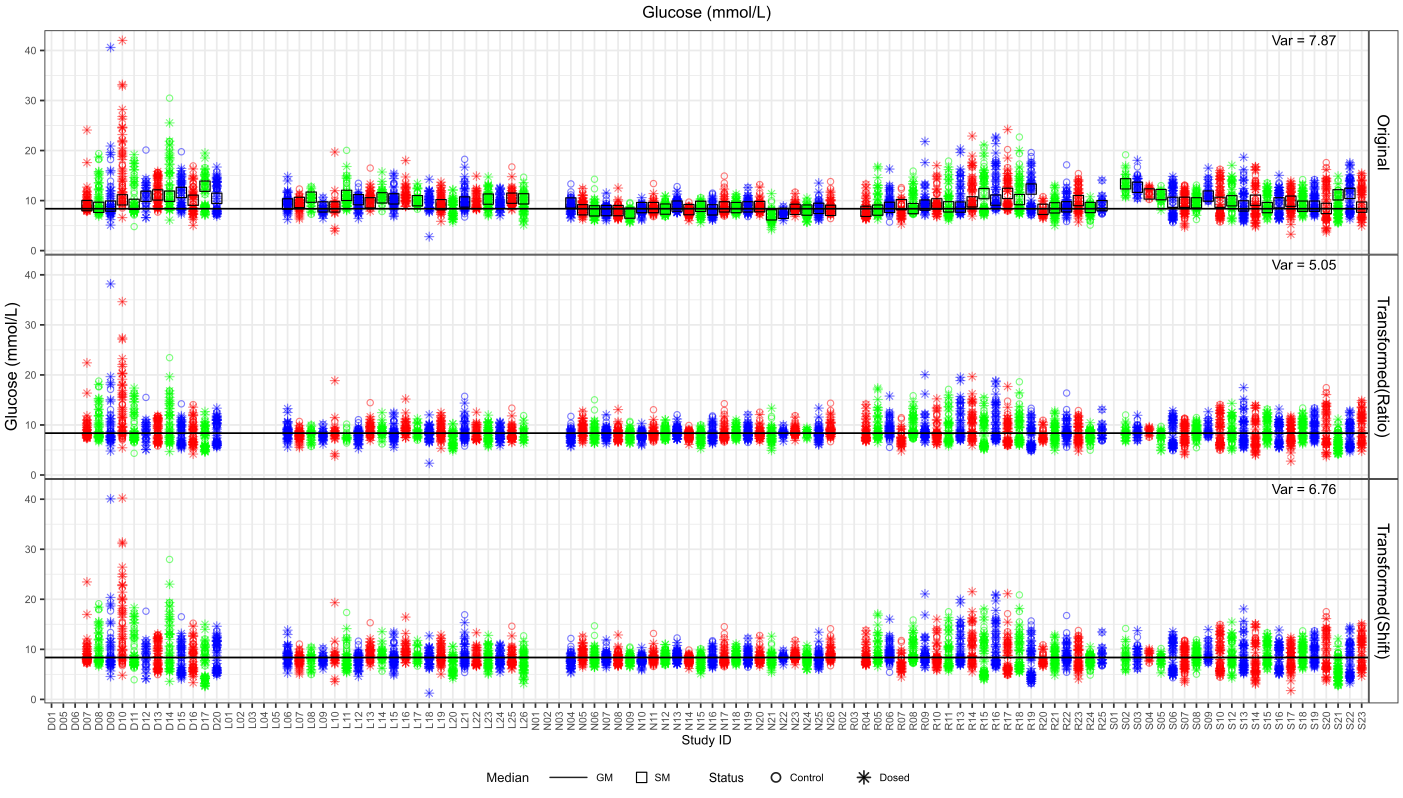


### Figure S2Fvi.

Glucose before (Original) and after batch corrections (Ratio and Shift). The total variance is displayed in the top right corner of each plot facet. Studies alternate colour for visualisation purposes.


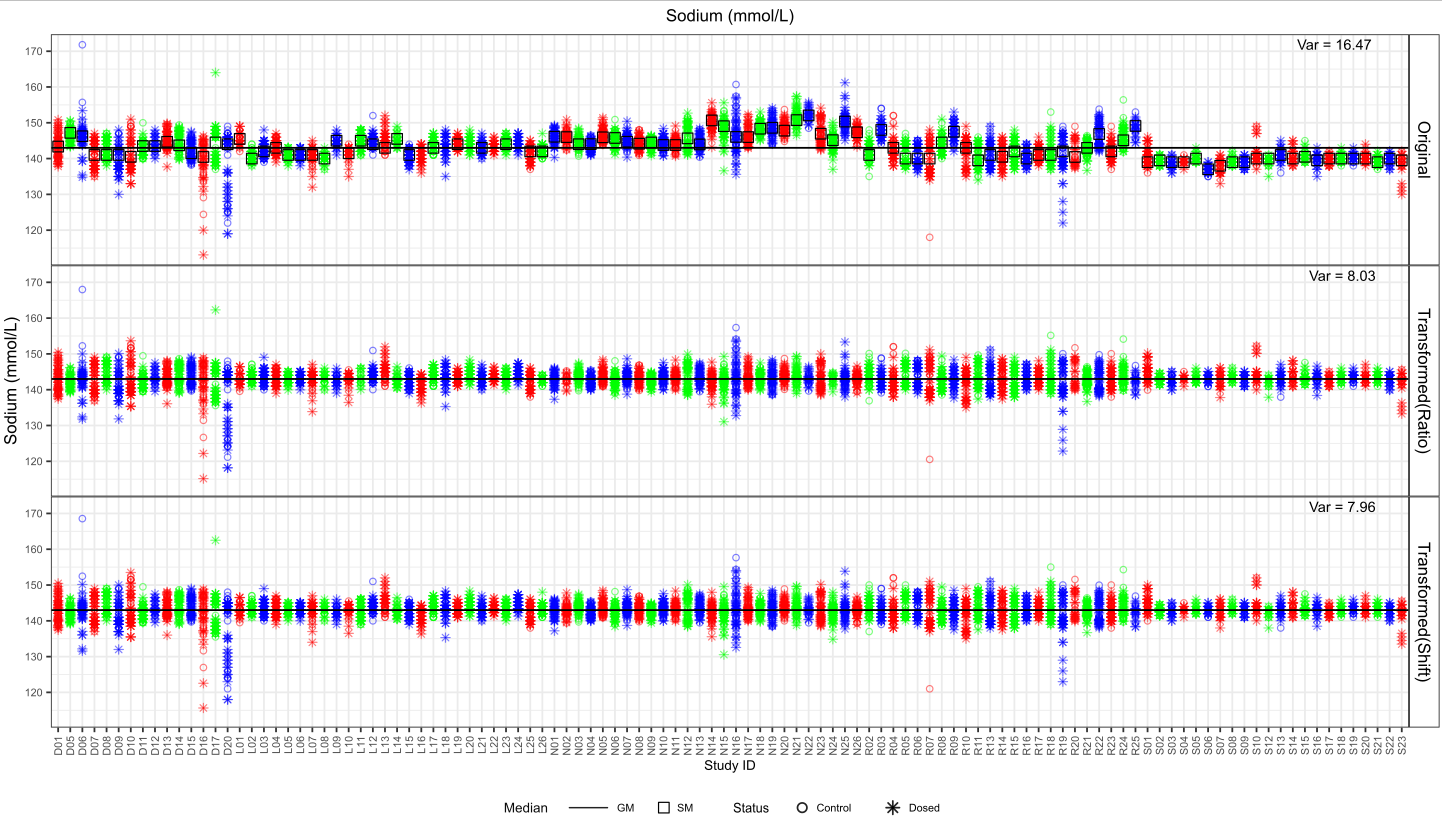


### Figure S2Fvii.

Sodium before (Original) and after batch corrections (Ratio and Shift). The total variance is displayed in the top right corner of each plot facet. Studies alternate colour for visualisation purposes.


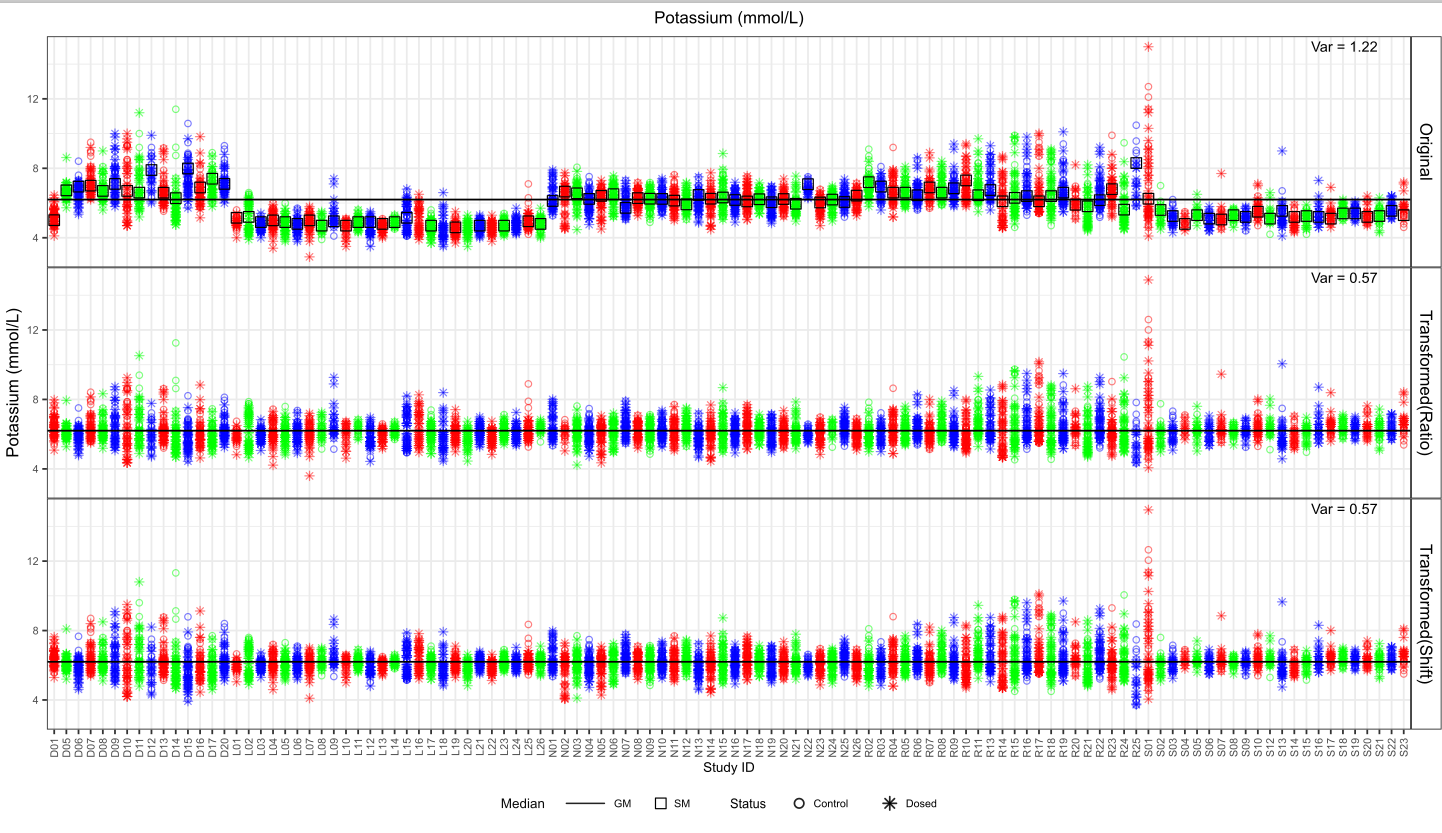


### Figure S2Fviii.

Potassium before (Original) and after batch corrections (Ratio and Shift). The total variance is displayed in the top right corner of each plot facet. Studies alternate colour for visualisation purposes.


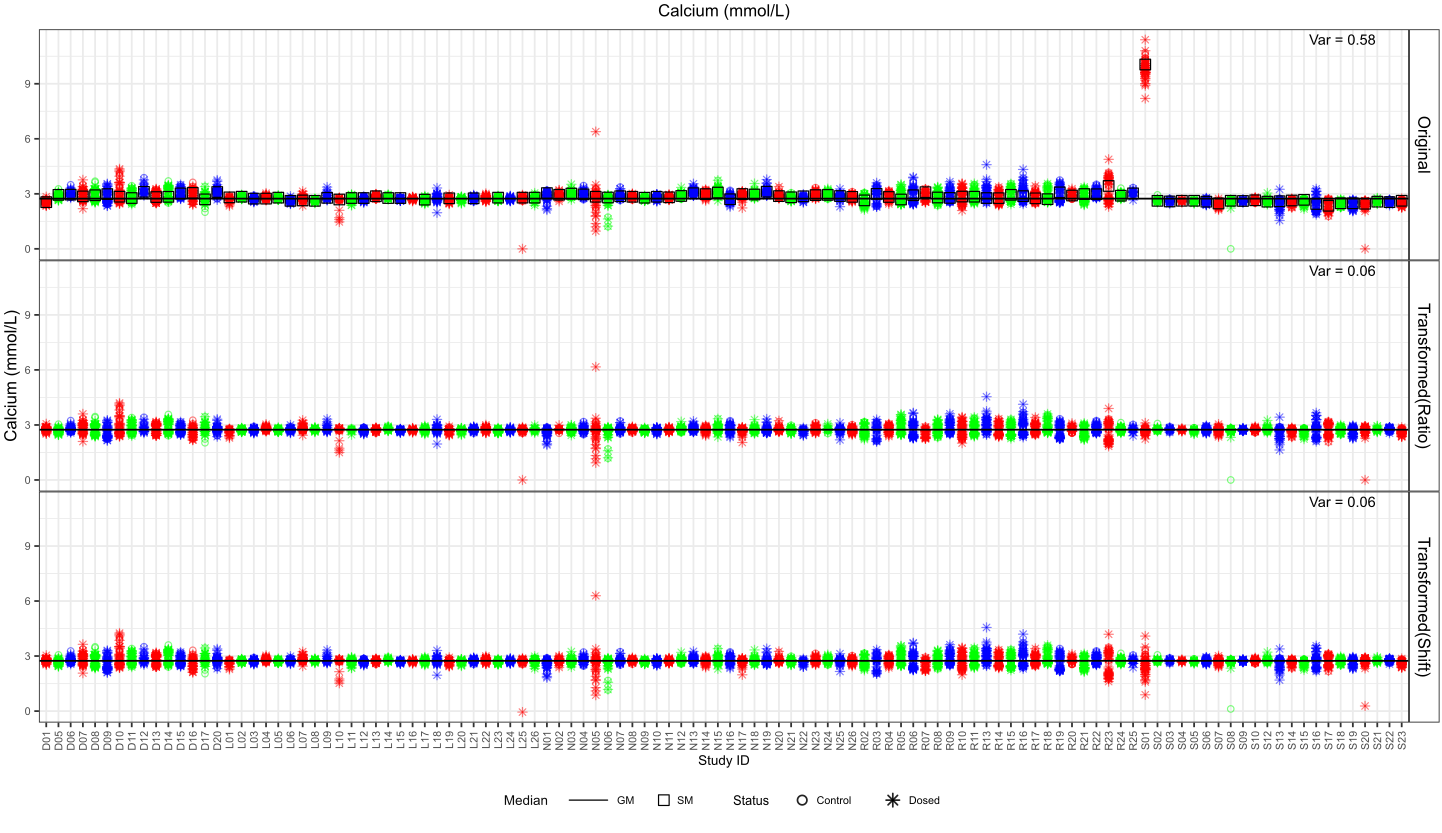


### Figure S2Fix.

Calcium before (Original) and after batch corrections (Ratio and Shift). The total variance is displayed in the top right corner of each plot facet. Studies alternate colour for visualisation purposes.


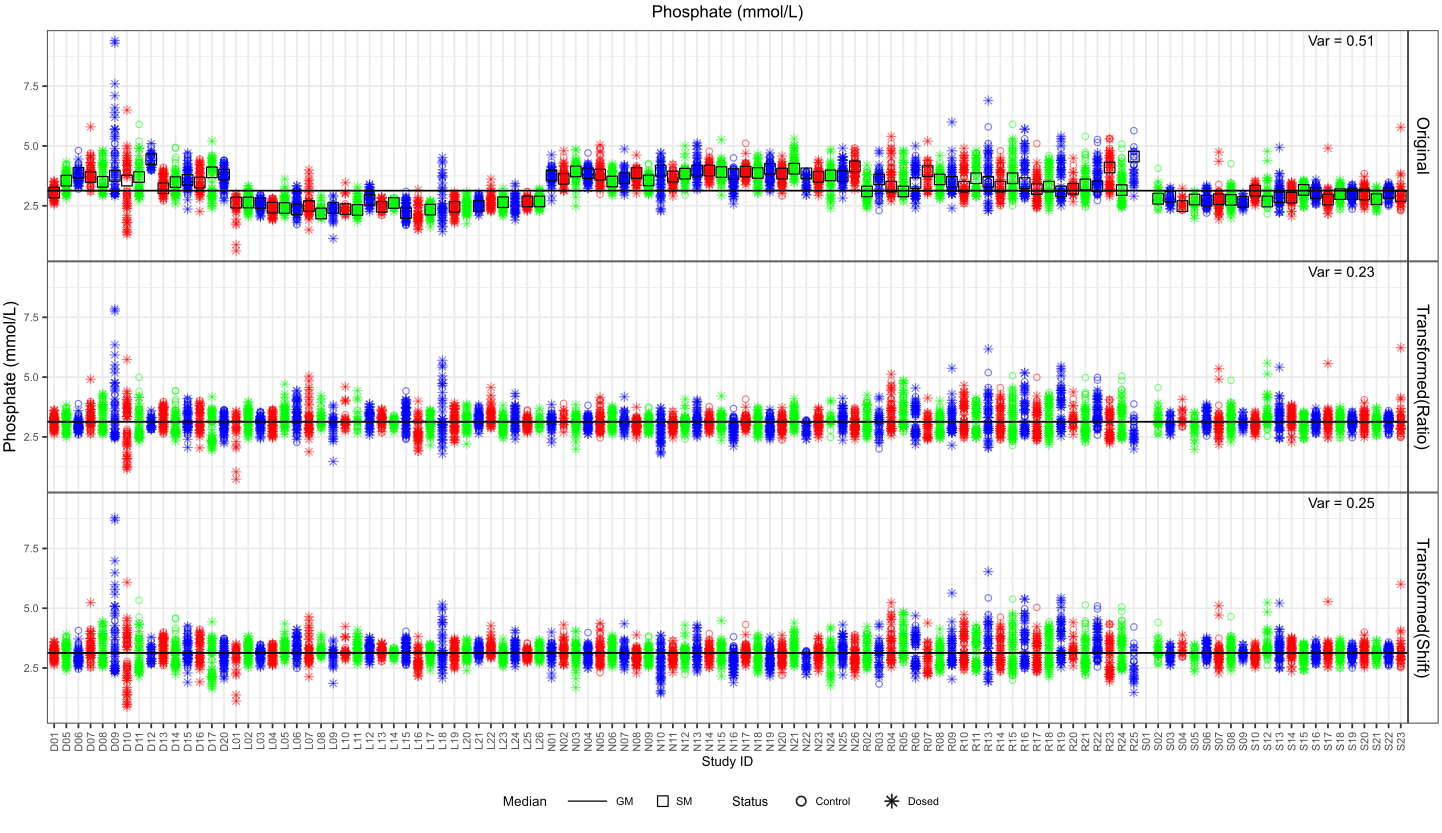


### Figure S2Fx.

Phosphate before (Original) and after batch corrections (Ratio and Shift). The total variance is displayed in the top right corner of each plot facet. Studies alternate colour for visualisation purposes.


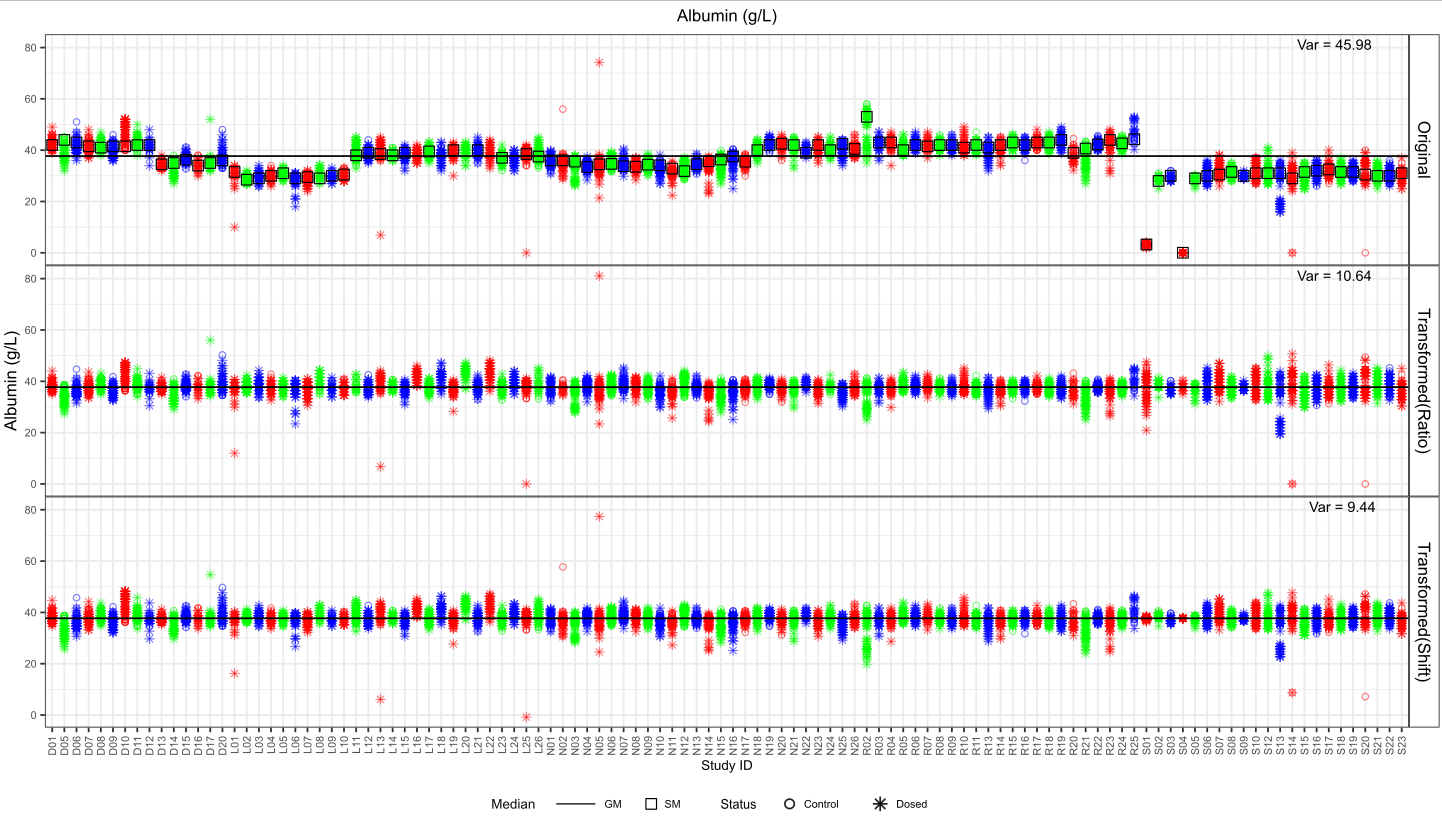


### Figure S2Fxi.

Albumin before (Original) and after batch corrections (Ratio and Shift). The total variance is displayed in the top right corner of each plot facet. Studies alternate colour for visualisation purposes.


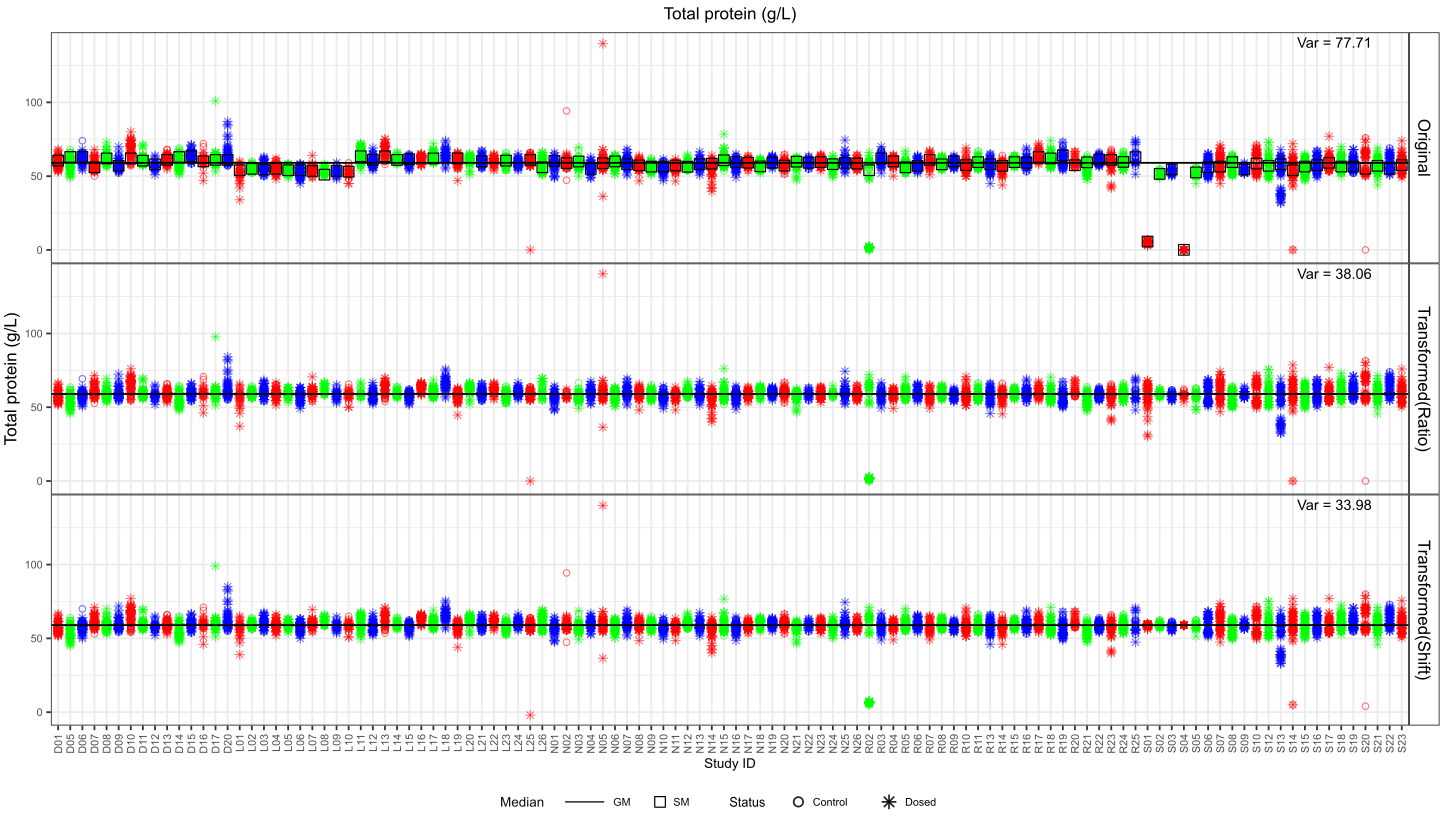


### Figure S2Fxii.

Total protein before (Original) and after batch corrections (Ratio and Shift). The total variance is displayed in the top right corner of each plot facet. Studies alternate colour for visualisation purposes.


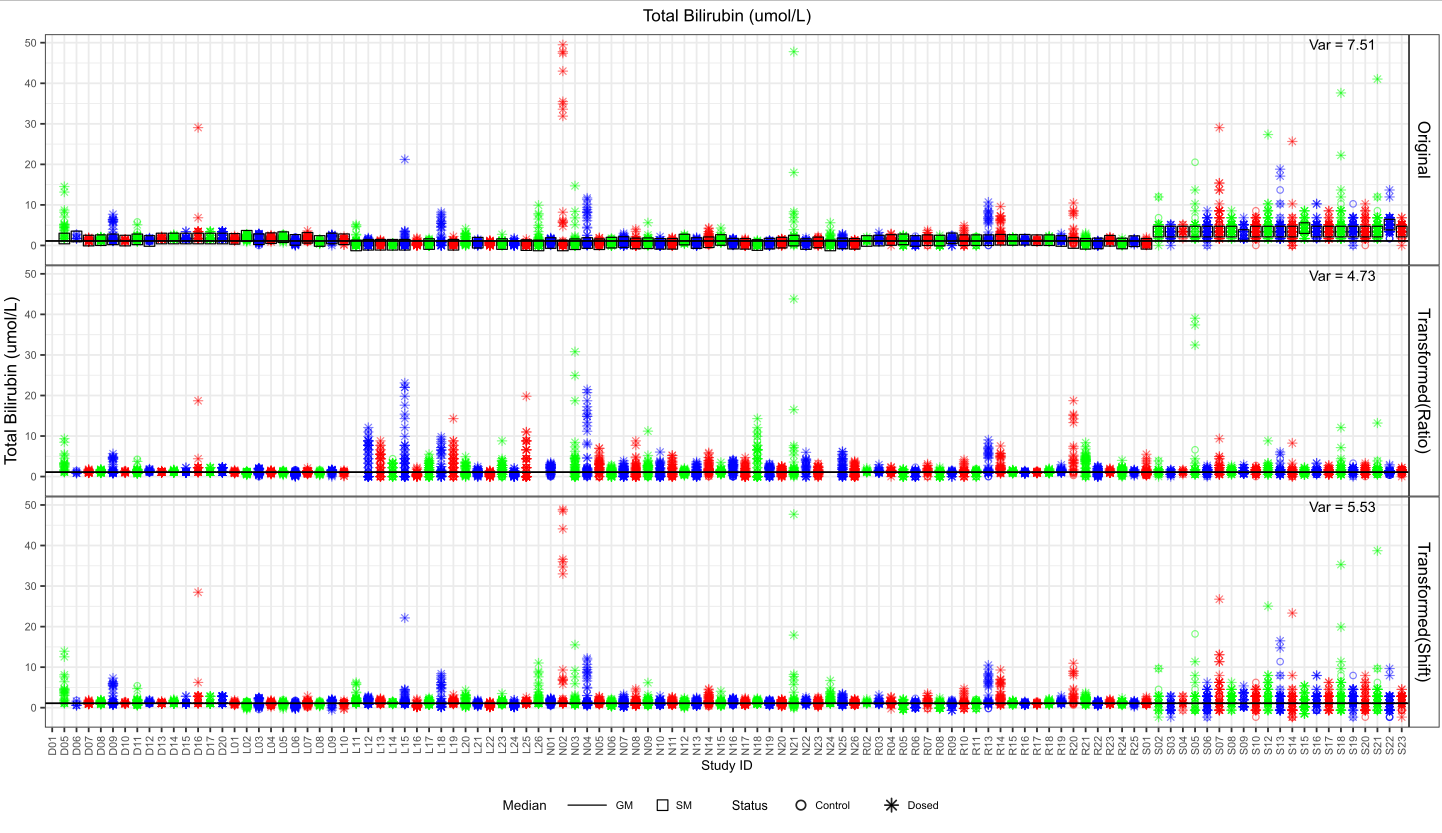


### Figure S2Fxiii.

Total bilirubin before (Original) and after batch corrections (Ratio and Shift). The total variance is displayed in the top right corner of each plot facet. Studies alternate colour for visualisation purposes.

**
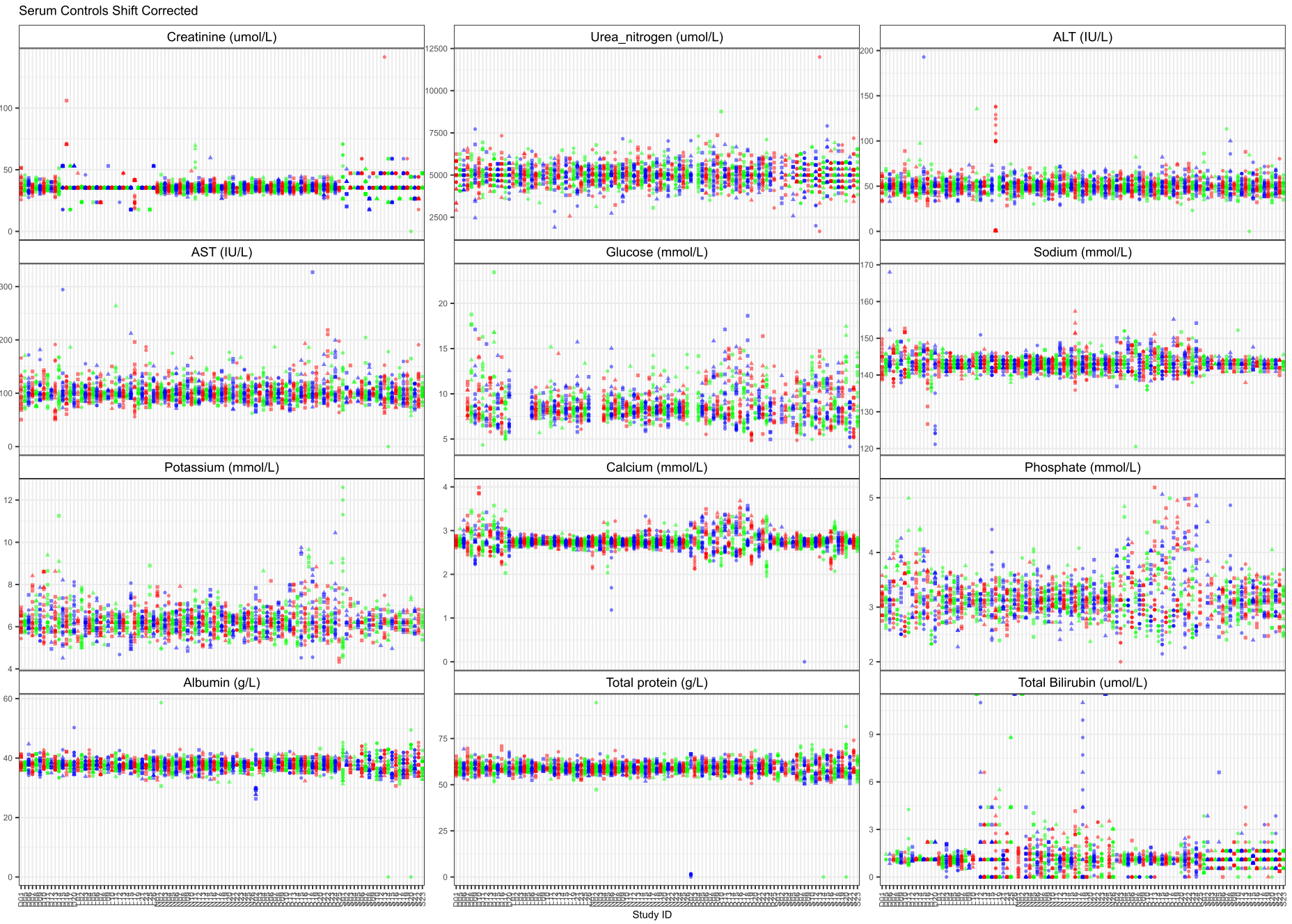
**

### Figure S2G.

Serum Control data after shift correction for all variables.

Both corrections were mostly effective at reducing the variance of the controls, with the ratio correction outperforming shift only once for glucose in **Figure S2Fi.** Though the variance reduction (**Figure S2Fi.**) and improvement of batch effects is similar for both, the shift correction better maintained the biologically relevant peaks in **Figure S2Fii.- Figure S2Fxiii.** For this reason, the shift correction was selected.

Post batch correction, the data was re-examined for negative values, which were changed to missing. The final sparsity is displayed in **Figure S2G**.


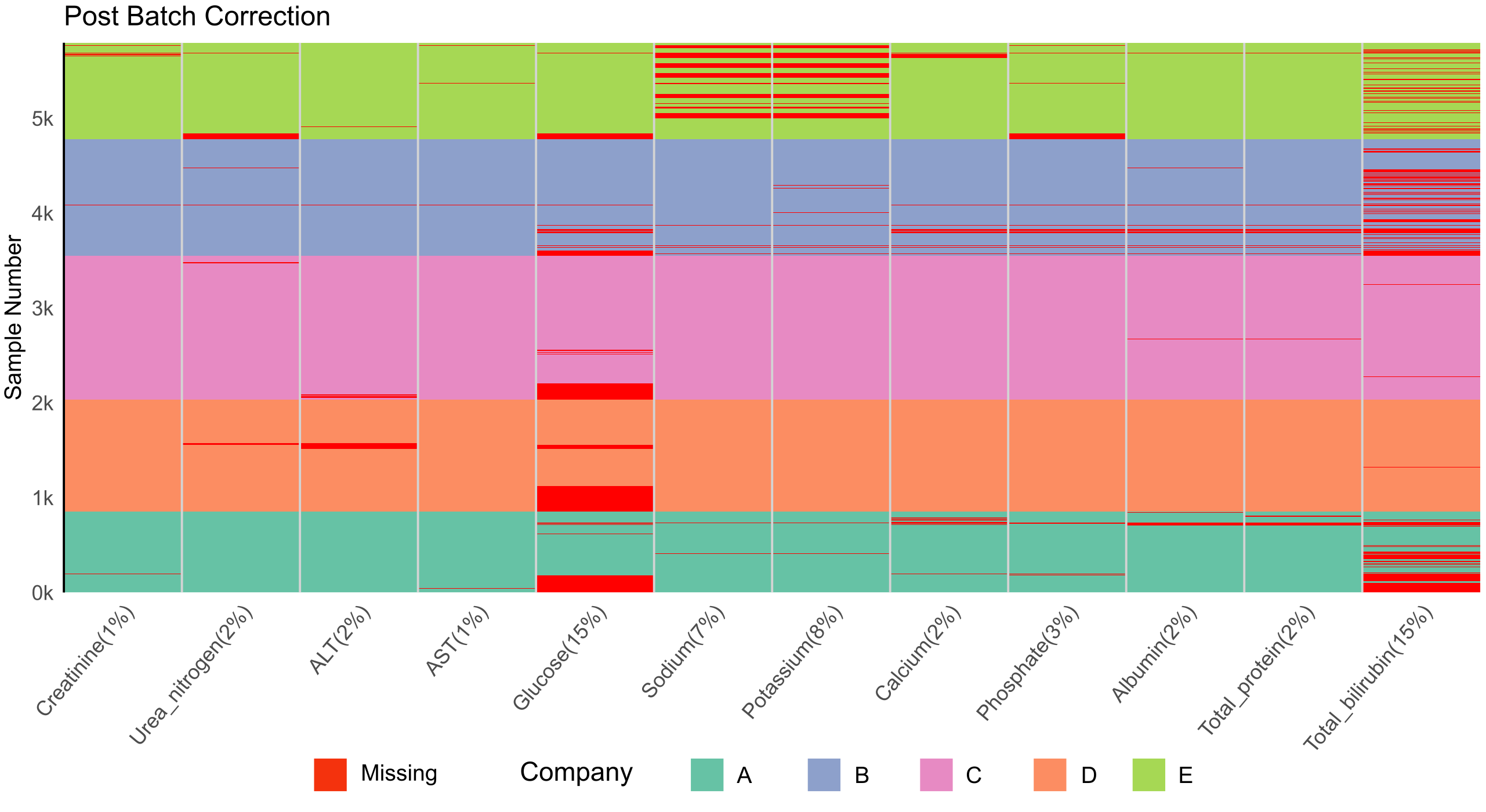


### Figure S2H.

The sparsity of serum parameters post-batch correction with biologically impossible values removed. The y axis depicts the sample number while the x axis dictates the variable and percentage missing as variable (%).

In ascending order, the following number of negative values required to be changed to missing post shift batch correction, 1 total protein, calcium and albumin, 2 creatinine, 9 AST, 231 total bilirubin and 251 ALT.

### Table S1.

List of COMET serum parameters with missing proportions, a total of 5,796 samples are present post data curation.

| **Serum parameter** | **Number of Missing Values** | **Proportion Missing (%)** |
| --- | --- | --- |
| Creatinine | 49 | 0.81 |
| Urea Nitrogen | 182 | 3.00 |
| ALT | 268 | 4.12 |
| AST | 53 | 0.87 |
| Glucose | 914 | 15.1 |
| Sodium | 496 | 8.18 |
| Potassium | 520 | 8.57 |
| Calcium | 134 | 2.21 |
| Phosphate | 175 | 2.88 |
| Albumin | 137 | 2.26 |
| Total Protein | 130 | 2.14 |
| Total Bilirubin | 943 | 15.5 |

### Table S2

Performance metrics.

| Metric | Purpose |
| --- | --- |
| Internal | |
| Normalised Root Mean Square Error (NRMSE) | Measures accuracy (Osman *et al.*, 2018; Emmanuel *et al.*, 2021; Jadhav *et al.*, 2019) compensating for differing scales of variables (Jadhav *et al.*, 2019) but is sensitive to extreme values (Chai and Draxler, 2014) and neglects uncertainty and potential bias (van Buuren, 2018). |
| Mean Absolute Error (MAE) | Not influenced by extreme values and serves as an improved precision measure compared to mean bias (Emmanuel *et al.*, 2021; Slade and Naylor, 2020). |
| External | |
| Bias (existing relationships) | Evaluates if results improve post-imputation and assesses potential bias introduced by imputed data. A known linear relationship in the data is used to find the gradient when using both CCA and imputed data (Sterne *et al.*, 2009). It assesses which slope is closest to the original. The difference between the original slope (Slope_Original_) and the changed slope (Slope_Changed_, imputed or complete cases only) measures bias (Penone *et al.*, 2014). For example, creatinine versus urea nitrogen and AST versus ALT are used to assess bias since both relationships have a strong correlation that should be preserved after imputation (Inaguma *et al.*, 2018). |
| Distribution | Determines if data integrity is maintained post-imputation. Changes in distributional features (shape, peak position, etc.) post-imputation may not indicate poor performance, but large differences require further assessment. Empirical density plots highlight changes in the distribution between the original and imputed data (Nguyen *et al.*, 2017; Sterne *et al.*, 2009; Lee and Carlin, 2017). Half-violin plots and boxplots compare the observed and imputed data distributions (Nguyen *et al.*, 2017; Sterne *et al.*, 2009). |


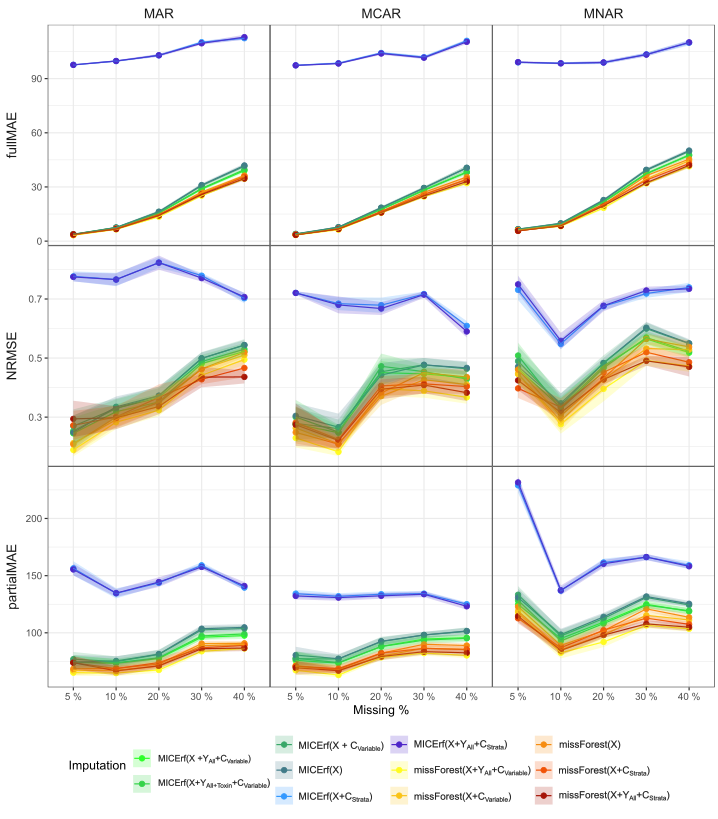


### Figure S3.

Internal performance metrics of imputation; NRMSE, full MAE and partial MAE for varying proportions of missing data for all three types of missingness A) MCAR B) MAR and C) MNAR using imputation methods missForest and MICErf. The solid line and points represent the mean values averaged over 20 iterations, while the shadow denotes the confidence interval.


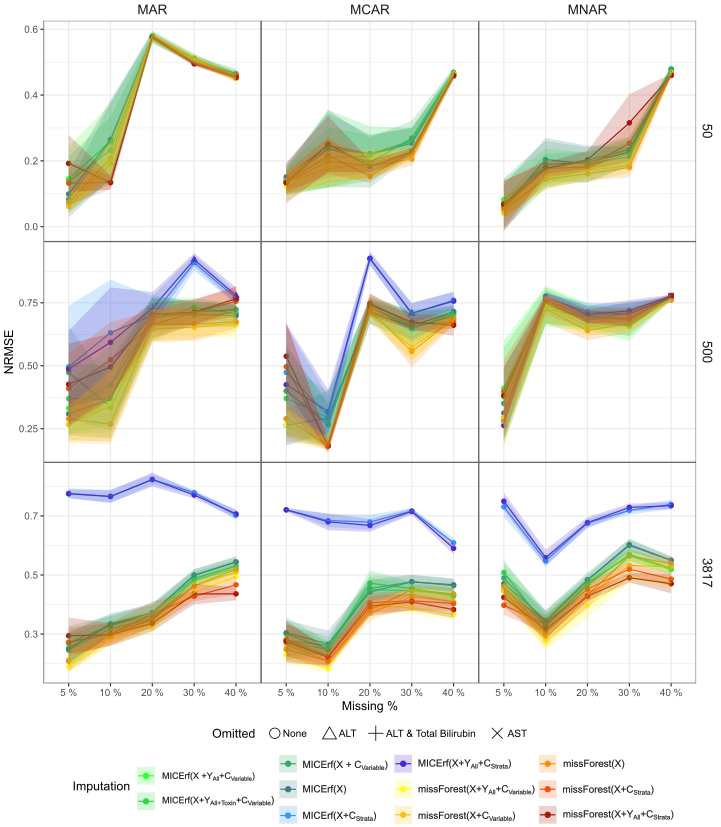


### Figure S4.

Internal performance metric NRMSE for different sample sizes 50, 500 and 2,769 under MCAR, MAR and MNAR for missing proportions 5, 10, 20, 30 and 40%. The points and solid line represent the mean values over 20 iterations while the shadow denotes the confidence interval. The stratified MICErf are completely absent from the sample size 50, and incomplete for a sample size of 500. Some points for the 500 sample size stratified MICErf are differing shapes to denote which serum parameters were omitted from the calculation due to MICErf being unable to impute them.


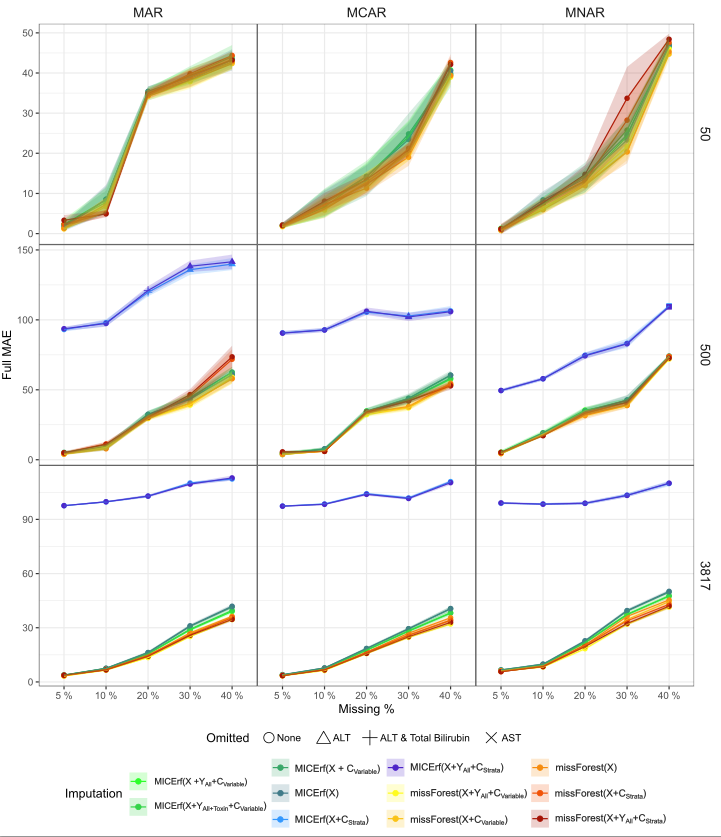


### Figure S5.

Internal performance metric full MAE for different sample sizes 50, 500 and 3,817 under MCAR, MAR and MNAR for missing proportions 5, 10, 20, 30 and 40%. The points and solid line represent the mean values over 20 iterations while the shadow denotes the confidence interval. The stratified MICErf are completely absent from the sample size 50, and incomplete for a sample size of 500. Some points for the 500 sample size stratified MICErf are differing shapes to denote which serum parameters were omitted from the calculation due to MICErf being unable to impute them.


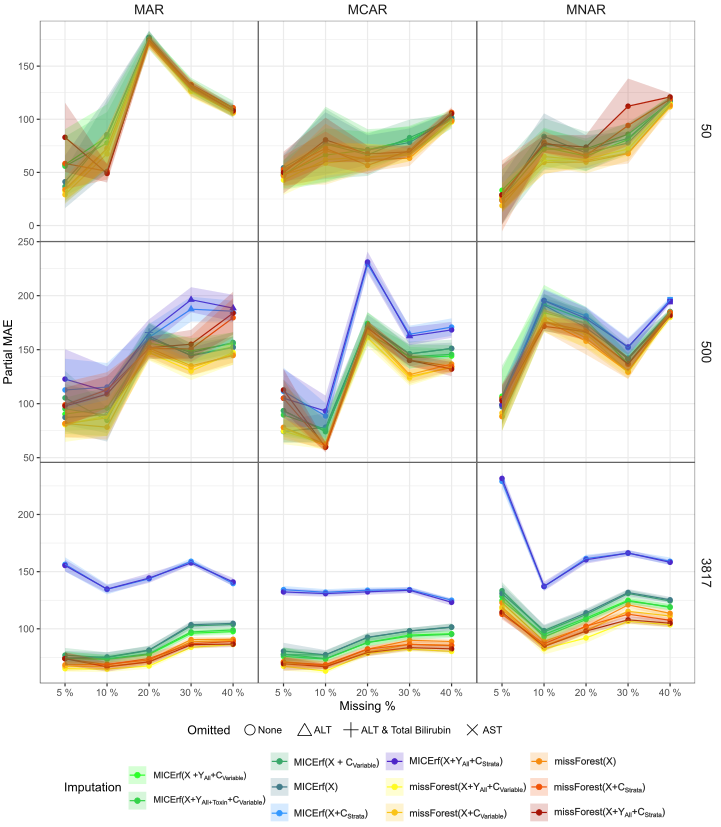


### Figure S6.

Internal performance metric partial MAE for different sample sizes 50, 500 and 3,817 under MCAR, MAR and MNAR for missing proportions 5, 10, 20, 30 and 40%. The points and solid line represent the mean values over 20 iterations while the shadow denotes the confidence interval. The stratified MICErf are completely absent from the sample size 50, and incomplete for a sample size of 500. Some points for the 500 sample size stratified MICErf are differing shapes to denote which serum parameters were omitted from the calculation due to MICErf being unable to impute them.


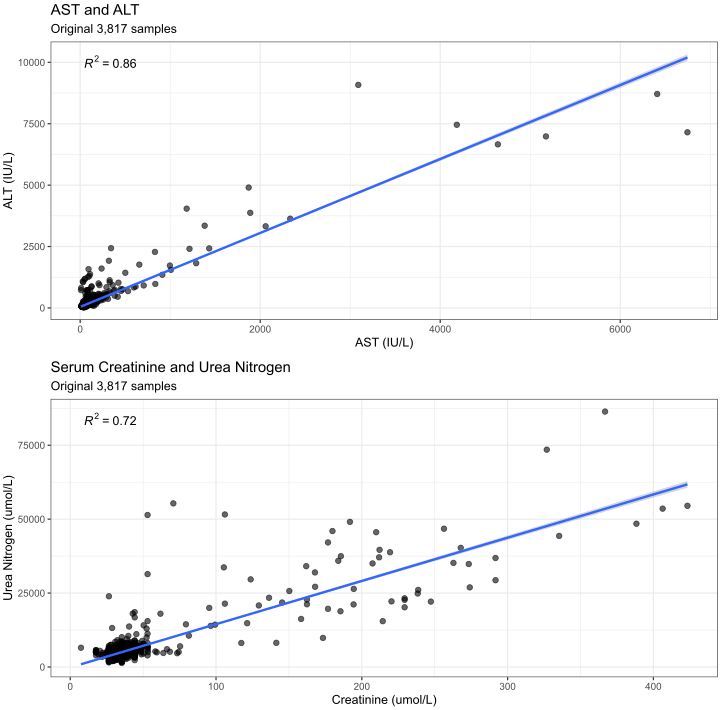


### Figure S7.

A graphical display of the two highest correlated relationships among all combinations for serum variables.


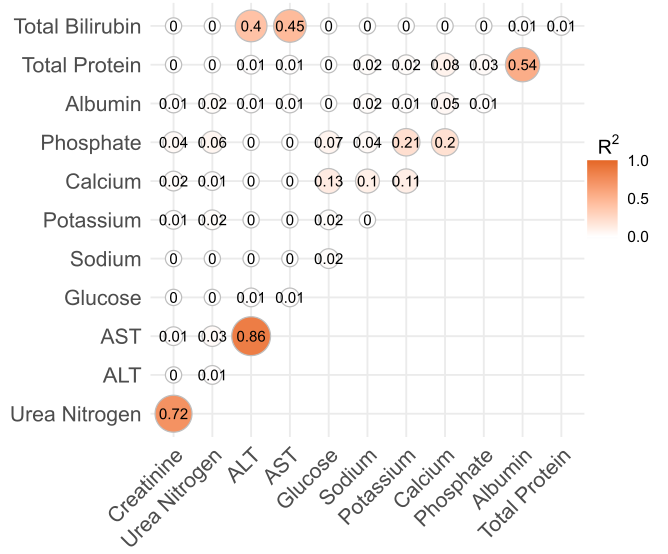


### Figure S8.

Correlation plot for all variables. Urea nitrogen vs Creatinine and ALT vs AST were selected as the strongest relationships to use for bias testing.


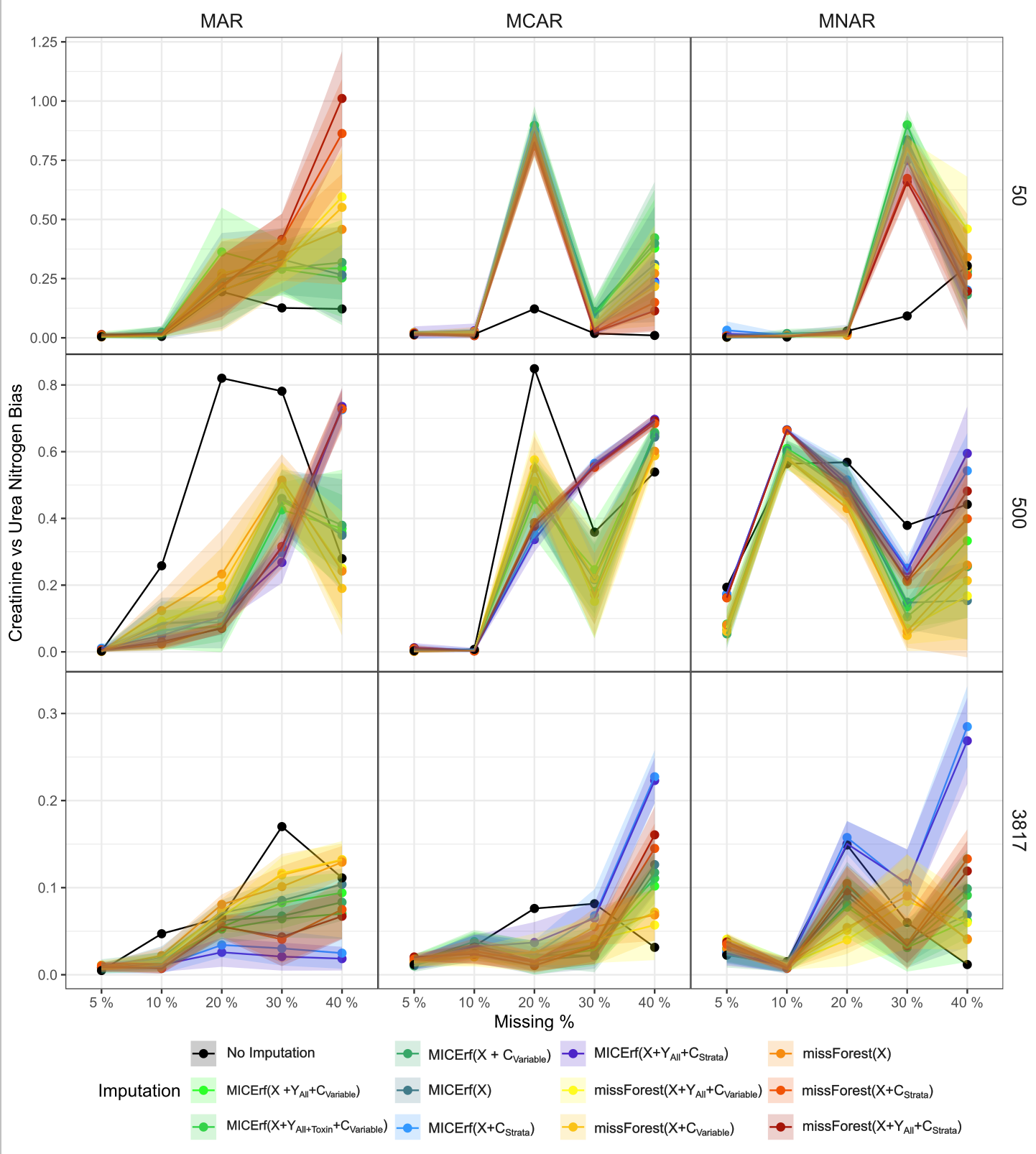


### Figure S9.

Imputation bias between creatinine versus urea nitrogen for different sample sizes 50, 500 and 3,817 under MCAR, MAR and MNAR for missing proportions 5, 10, 20, 30 and 40%. The points and solid line represent the mean values over 20 iterations while the shadow denotes the confidence interval. The stratified MICErf are completely absent from the sample size 50. The incomplete imputation for a sample size of 500 did not affect Creatinine or Urea Nitrogen.


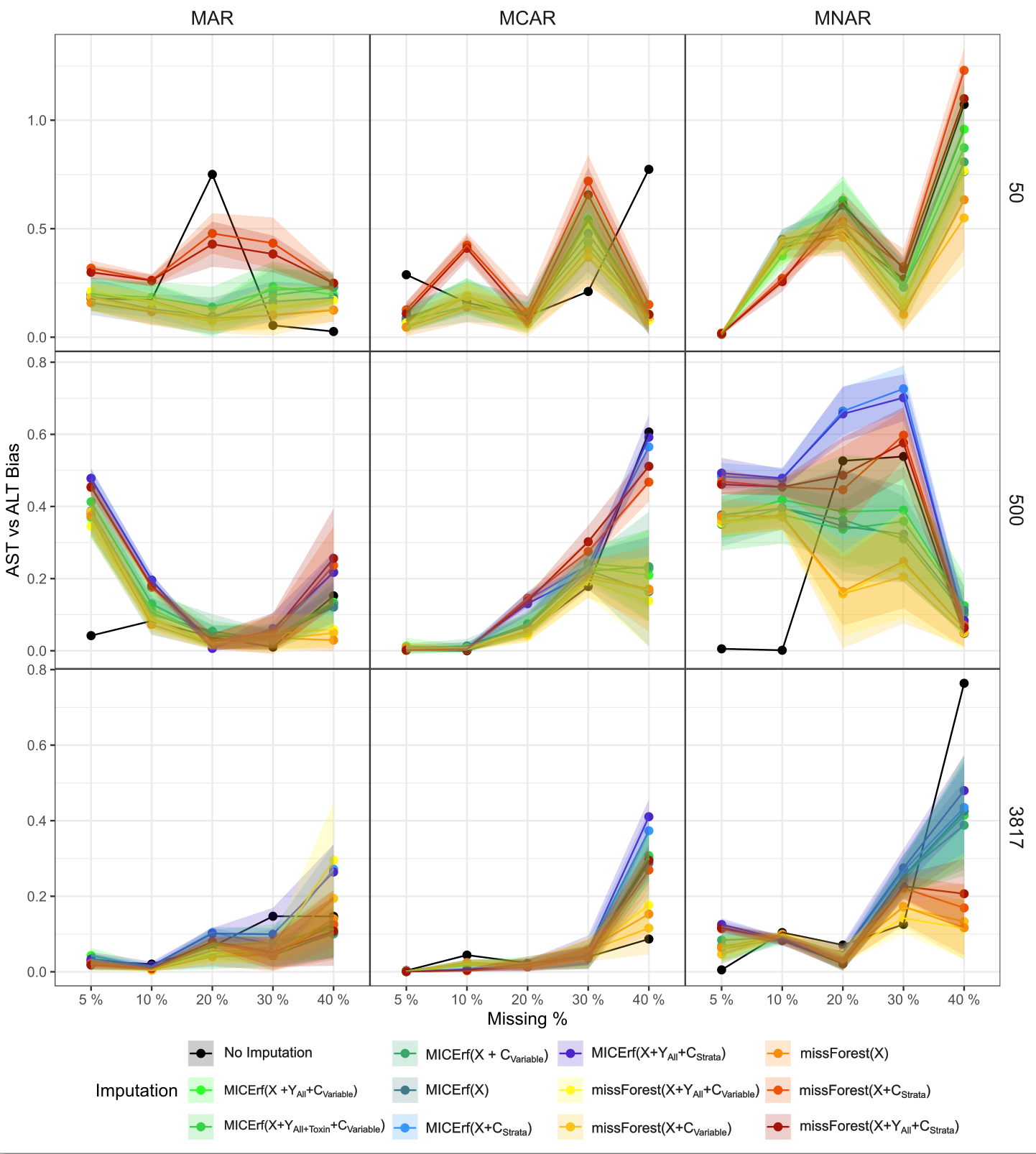


### Figure S10.

Imputation bias between AST versus ALT for different sample sizes 50, 500 and 3,817 under MCAR, MAR and MNAR for missing proportions 5, 10, 20, 30 and 40%. The points and solid line represent the mean values over 20 iterations while the shadow denotes the confidence interval. The stratified MICErf are completely absent from the sample size 50. The incomplete imputation for a sample size of 500 omitted the points that were unable to be imputed for AST or ALT.


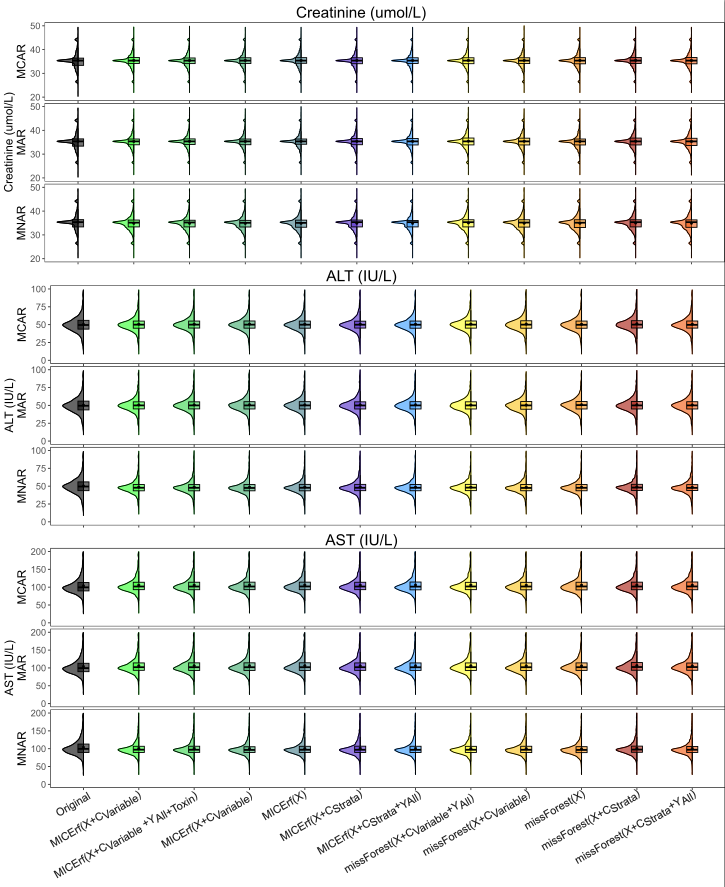


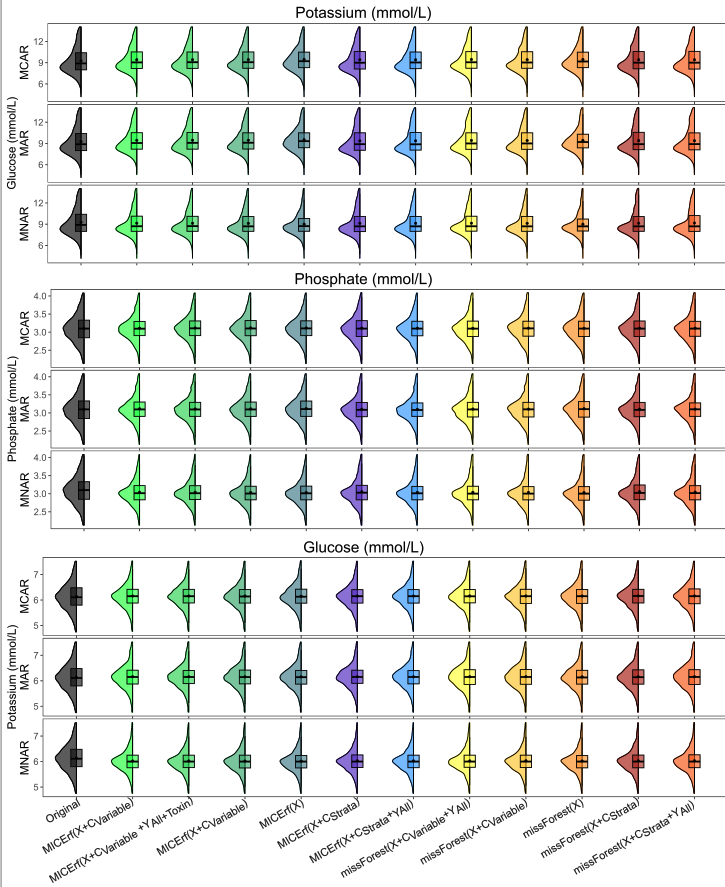


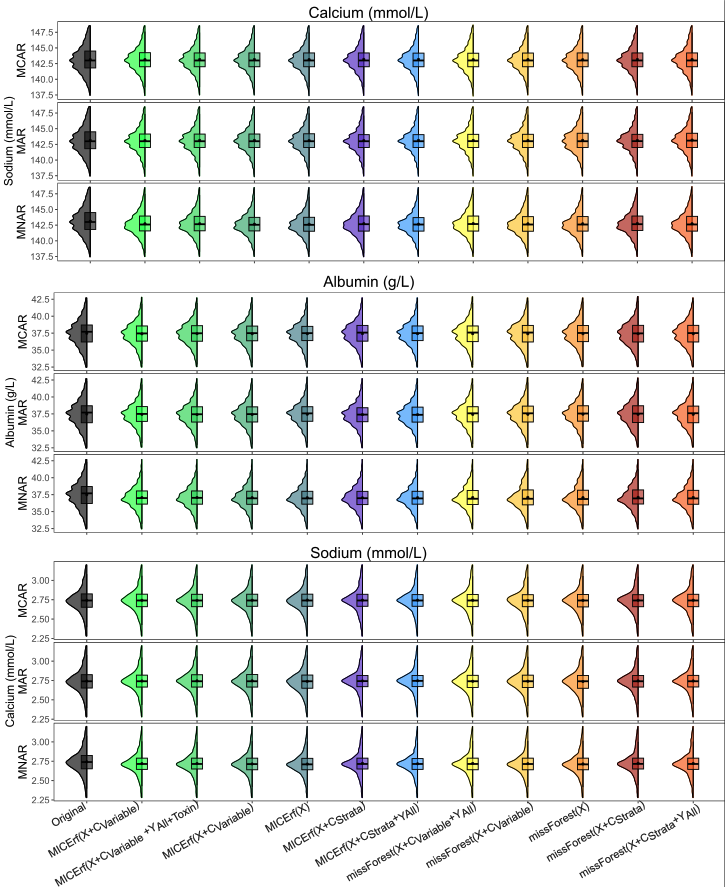

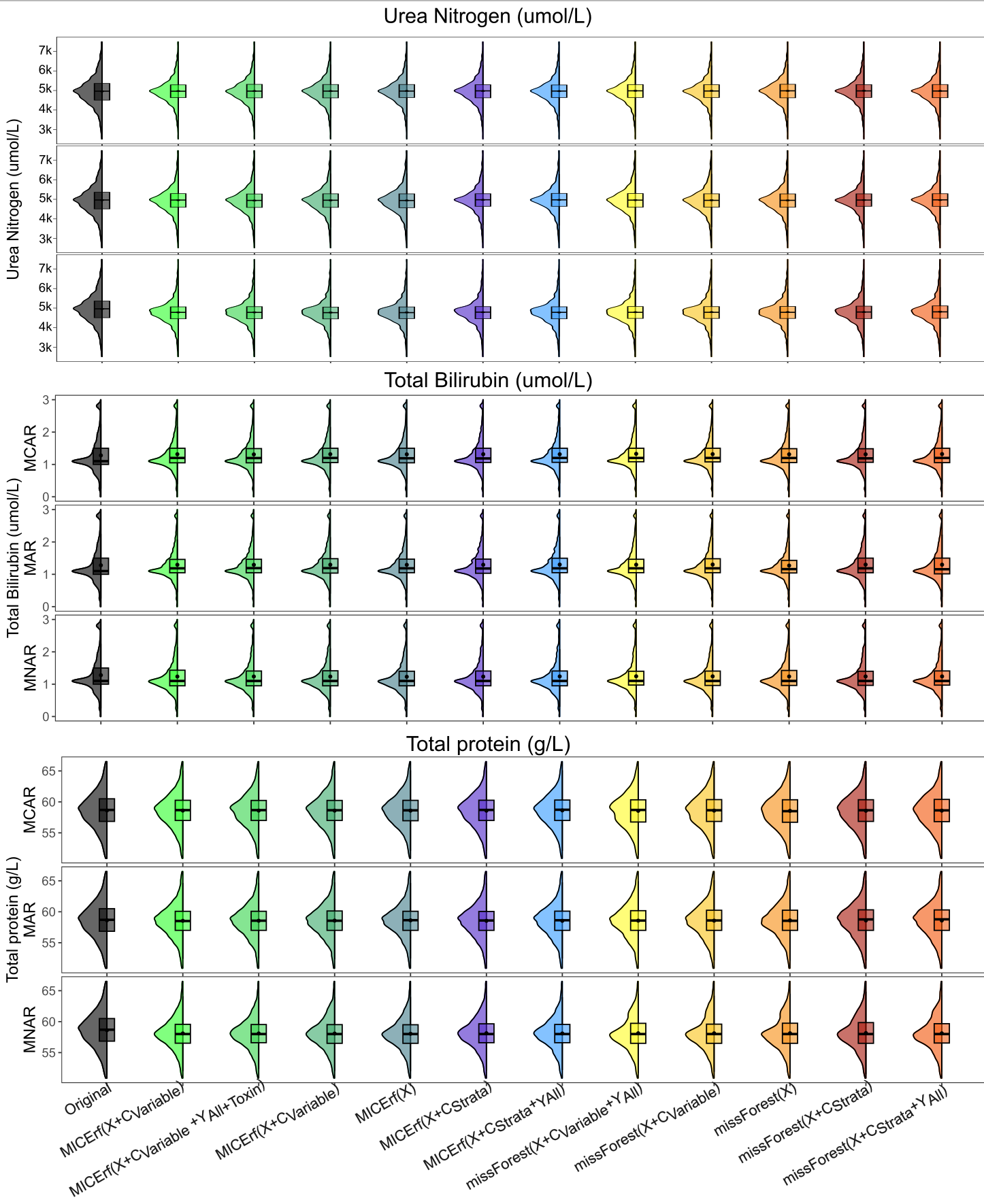


### Figure S11.

##### Half violin box plots of the median ± 2 standard deviations to illustrate the distribution of each of the remaining clinical serum parameters at 3,817 complete cases (Original in black) compared to post imputation. Displayed for 40 % missing for all three types of missingness MAR, MNAR, and MCAR using imputation methods, MICErf and missForest.

#####

The distributions in **Figure S11**. of values post imputation for 40% missing are visibly consistent with the original (using the complete data) except for MNAR. The stratification of variable inclusion had minimal effect on the distribution.
